# Exploring Nanocluster Potential Energy Surfaces via Deep Reinforcement Learning: Strategies for Global Minimum Search

**DOI:** 10.1021/acs.jpca.4c04416

**Published:** 2024-10-14

**Authors:** Rajesh K. Raju

**Affiliations:** † National Research Council Canada, Clean Energy Innovation (CEI) Research Centre, Mississauga, Ontario L5K 1B4, Canada; ‡ School of Chemistry, University of Birmingham, Birmingham B15 2TT, U.K.

## Abstract

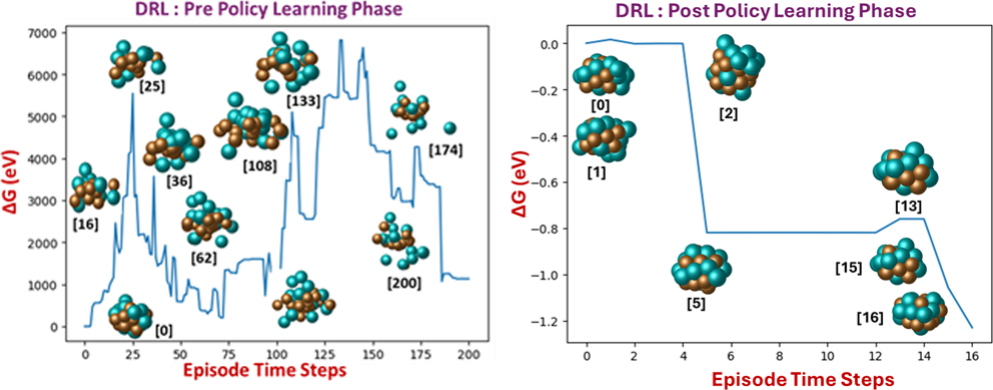

The search for global minimum (GM) configurations in nanoclusters is complicated by intricate potential energy landscapes replete with numerous local minima. The complexity of these landscapes escalates with increasing cluster size and compositional diversity. Evolutionary algorithms, such as genetic algorithms, are hampered by slow convergence rates and a propensity for prematurely settling on suboptimal solutions. Likewise, the basin hopping technique faces difficulties in navigating these complex landscapes effectively, particularly at larger scales. These challenges highlight the need for more sophisticated methodologies to efficiently scan the potential energy surfaces of nanoclusters. In response, our research has developed a novel deep reinforcement learning (DRL) framework specifically designed to explore the potential energy surfaces (PES) of nanoclusters, aiming to identify the GM configurations along with other low-energy states. This study demonstrates the framework’s effectiveness in managing various nanocluster types, including both mono- and multimetallic compositions, and its proficiency in navigating complex energy landscapes. The model is characterized by remarkable adaptability and sustained efficiency, even as cluster sizes and feature vector dimensions increase. The demonstrated adaptability of DRL in this context underscores its considerable potential in materials science, particularly for the efficient discovery and optimization of novel nanomaterials. To the best of our knowledge, this is the first DRL framework designed for the GM search in nanoclusters, representing a significant innovation in the field.

## 1 Introduction

Nanoclusters, characterized by their distinct electronic, optical, magnetic, and chemical attributes, exhibit properties that diverge significantly from those observed in their bulk counterparts.^1−6^ Moreover, nanoalloys, also known as alloy nanoclusters, have gained significant interest in the field of nanocatalysis and exhibit unique catalytic properties due to their distinct physical and chemical characteristics, differing significantly from pure metal nanoclusters.^7−9^ The intricacies of these properties are predominantly influenced by factors such as size, shape, and composition. A comprehensive understanding of these nuances is imperative for the bespoke tailoring of materials for specified functions. Modeling plays a pivotal role in this realm, enabling scientists to not only comprehend but also to predict and design novel materials imbued with desired characteristics. In the context of catalysis, nanoclusters are esteemed for their exceptional surface-to-volume ratio and distinctive electronic properties.

The pursuit of identifying the global minimum (GM) in nanoclusters presents a multitude of challenges, primarily stemming from the intricate nature of these systems. The plethora of possible atomic configurations in nanoclusters renders the exploration of their entire configuration space a daunting, computationally intensive, and time-consuming endeavor. Furthermore, numerous configurations exhibit energy values that are closely aligned, thereby complicating the identification of the most stable configuration. Due to all these, the potential energy surface (PES) of nanoclusters is typically rugged and intricate and this complexity often poses significant challenges to optimization algorithms in their quest to locate the GM.

In the endeavor to determine the GM configurations of nanoclusters, evolutionary algorithms like genetic algorithms (GAs) and the basin hopping (BH) method are frequently employed, yet they exhibit significant limitations.^10−14^ GAs face challenges such as slow convergence rates, a propensity for premature convergence to suboptimal solutions due to diminishing population diversity, and difficulties navigating complex energy landscapes, particularly with larger nanoclusters. The efficiency of GAs is critically dependent on the optimal selection of parameters, including population size and mutation rate. Moreover, these algorithms are susceptible to becoming trapped in local minima. Conversely, the BH method, which integrates Monte Carlo steps with local minimization procedures, is contingent on factors such as the choice of step size and the performance of the local minimization algorithm. This method can encounter difficulties in highly intricate landscapes, and its effectiveness diminishes as the size of nanoclusters increases, resulting in decreased efficiency and increased time consumption. This highlights an urgent need for further optimization and refinement of these methodologies to improve their effectiveness in the GM search for nanoclusters.

Previous work by Zhai and Alexandrova introduced a GPU-accelerated global optimization approach that combines deep neural network (DNN) fitting with limited-step density functional theory (DFT) optimization, significantly reducing the computational cost of full DFT local optimization for metal clusters, as demonstrated on Pt_9_ and Pt_13_ clusters.^15^ Similarly, Raju et al. developed an active learning genetic algorithm framework designed to accelerate the discovery of global minimum configurations for both pure and alloyed nanoclusters.^16^ Wang et al.^17^ accelerated the genetic algorithm (GA) search for aluminum nanoclusters using on-the-fly machine learning, while Hansen and colleagues^18^ employed a symmetry-constrained GA with a neural network potential to predict the energies of Pt–Ni nanoalloys. The GOFEE method developed by Bisbo and Hammer,^19^ an evolutionary algorithm enhanced by a machine-learned surrogate model and Bayesian statistics, efficiently identifies low-energy structures in complex energy landscapes described by first-principles methods, as demonstrated in studies on carbon clusters in both gas-phase and supported environments.

A crucial aspect of these evolutionary algorithms is the generation of configurations for evaluation. This task demands skillful creation of new configurations derived from existing geometries, with an aim for these new configurations to converge into novel local minima on the PES. Often, if the newly generated configurations for evaluation are too similar to existing geometries, they may converge into the same local minimum. Therefore, the effectiveness of these methods is largely reliant on the strategies adopted for generating innovative geometries for evaluations in evolutionary algorithms. This aspect underscores the need for advanced strategies in configuration generation to improve the success rate of locating GM in nanocluster research.

Addressing these challenges, this study introduces a novel approach utilizing deep reinforcement learning (DRL) for the PES scan of nanoclusters aimed at identifying GM configurations. This approach begins with random configurations, allowing the model to generate a diverse range of geometries. Such diversity is crucial for effectively scanning the PES and accurately locating GM configurations. This DRL-based approach represents a significant advancement in the field, offering a more efficient and effective means of exploring the complex landscape of nanocluster configurations and potentially overcoming the limitations of traditional evolutionary algorithms. The framework is versatile, applicable to both pure nanoclusters and alloyed nanoclusters comprising multiple metallic elements.

## 2 Methods

### 2.1 Machine Learning Model

Deep reinforcement learning (DRL) emerges as a proficient approach in navigating the intricate high-dimensional configuration search spaces arising from the multitude potential atomic arrangements. DRL’s inherent design makes it particularly suitable for dealing with high-dimensional and complex search spaces, such as those presented by nanoclusters. Recent developments in using reinforcement learning (RL) for optimizing molecular structures and chemical reaction pathways have shown its effectiveness in addressing intricate molecular design challenges.^20−27^

Traditional optimization methods might falter in the complex potential energy landscapes, where the sheer variety of possible atomic configurations creates a daunting challenge. This proficiency stems from DRL’s capacity to learn optimal policies through advanced neural networks. These networks are adept at processing extensive volumes of information with high efficiency. DRL distinguishes itself from many conventional optimization algorithms by its ability to refine its approach through direct interaction with the system under study. This interaction enables DRL to potentially uncover innovative strategies for locating the GM, strategies that are not pre-encoded in algorithmic rules.

**Figure 1:**
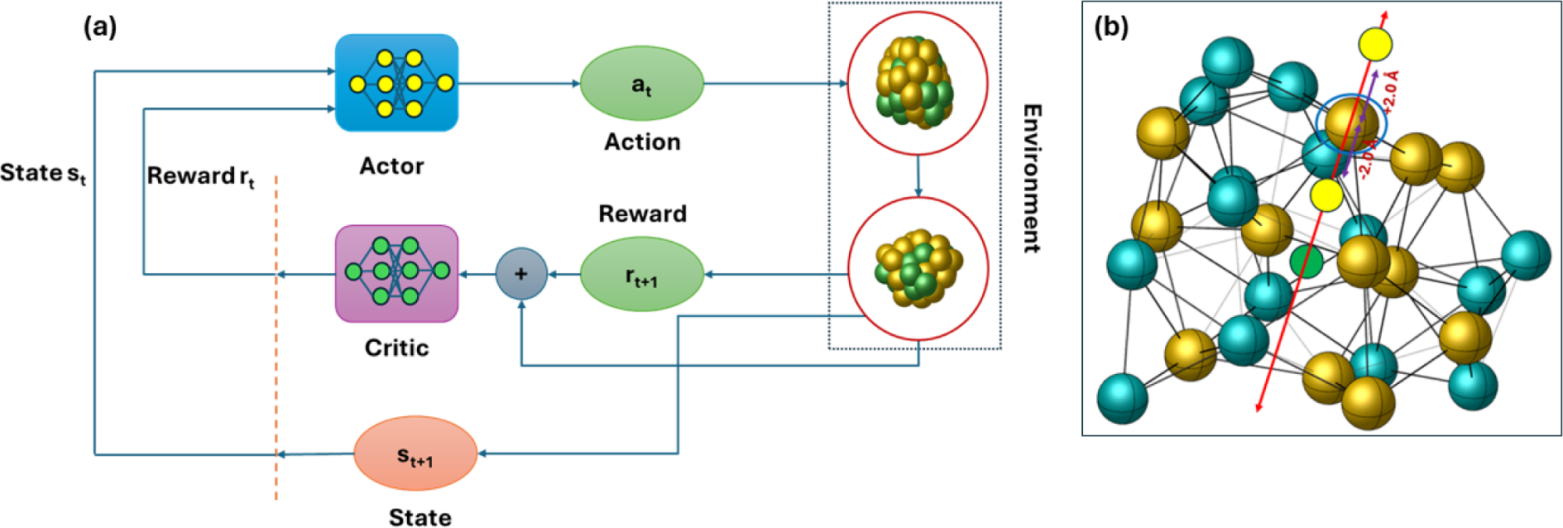
(a) Schematic representation of the DRL framework used in our work; (b) illustration of action step on cluster environment: visualization of an action sequence on a nanocluster, where the action consists of selecting a target atom (indicated in blue circle) during the initial action step , and subsequently relocating it to a new position (highlighted in yellow) at a distance of either +2.0 Å or −2.0 Å in the second action step . The direction of movement is determined by the vector connecting the atom’s initial position to the nanocluster’s center of mass (highlighted in green).

DRL, a subset of artificial intelligence, merges the principles of reinforcement learning (RL) and deep learning (DL). RL involves an agent that learns to make decisions through interactive trial-and-error engagement with an environment.^28^ This learning process is driven by feedback from the agent’s actions and experiences, in the form of positive or negative rewards, with the objective of maximizing the cumulative rewards from these actions. Figure 1a displays a schematic diagram of the DRL framework specifically developed for scanning the PES of nanoclusters.

In the specific context of nanocluster PES scan and GM search, DRL’s methodology allows the algorithm to learn and adapt its strategy dynamically, based on the feedback received from the outcomes of its actions. This dynamic adaptation facilitates the discovery of efficient pathways toward the GM, a task that is often challenging for traditional methods. A critical aspect of DRL algorithms is their designed capability to balance exploration and exploitation. Exploration refers to the investigation of new and potentially superior configurations, while exploitation focuses on refining and optimizing configurations that are already known to be effective. This balance is essential in the quest for the GM within a complex energy landscape. Through its adaptive learning mechanism and balance between exploration and exploitation, DRL offers a promising avenue for addressing the challenges posed by the GM search of nanoclusters, representing a significant advancement over traditional optimization techniques in this field.

The RL process is conceptualized as a loop outputting a sequence of states, actions, rewards. Following the Markov decision process (MDP), at each time step *t*, the RL agent select an action *a*_*t*_ from a set of possible actions, known as the action space *A*, given the current state *s*_*t*_ from the state space *S*. The environment, in response to the action, yields a reward *r*_*t*_ for the state-action pair  and transitions to the next state based on the transition probability . The agent learns a policy π_*t*_, which is essentially a probabilistic distribution over actions conditioned on the states. This policy, which maps the state space *S* to the action space *A* serves as the agent’s “brain,” guiding decision-making at each time step. The ultimate goal of the RL agent is to learn an optimal policy  that maximizes the expected cumulative reward , where γ is a discount factor balancing the importance of short-term and long-term rewards, and *T* represents the length of the episode.

In RL, a considerable challenge emerges in the form of the “curse of dimensionality,” which refers to the complications that arise when dealing with MDPs that have expansive, high-dimensional state spaces. To circumvent this issue, DRL integrates the robust capabilities of deep learning (DL) with the strategic framework of RL. This integration facilitates the handling of these high-dimensional spaces by employing neural networks to represent the decision-making policy, thereby enhancing the ability of the RL agent to interpret and act within complex environments. DRL has been applied successfully in various real-world scenarios, including robotic control, autonomous vehicles, and complex games. For our specific application, we implemented the trust region policy optimization (TRPO)^29^ within our DRL framework to efficiently optimize the policy. TRPO, an actor-critic network^30−32^ algorithm, amalgamates the benefits of both value-based and policy-based methods. The actor network determines the actions for given state embeddings, while the critic network assesses the quality of these actions. The actor’s learning is grounded in the policy gradient approach, while the critic evaluates actions by computing the value function. This actor-critic approach mitigates the variance of the policy gradient by using the critic’s value function as a baseline. TRPO, uniquely, integrates the actor-critic approach with a trust region constraint to regulate policy updates during training, thus enhancing training stability by preventing excessively large policy changes in a single step.

In our research, we delineate the formulation of a RL problem for exploring the PES of nanoclusters, aimed at identifying GM configurations. This formulation encompasses the definition of the state space (S), action space (A), and reward function, each tailored to the specific challenges of nanocluster PES analysis.

#### 2.1.1 State Space Definition and Encoding

The state space (S) represents the set of all possible environmental states, with each state being an embodiment of the environment’s current condition. In our study, the state space is defined by the configurations of nanoclusters. To encode these configurations for machine learning (ML) training, we employed atom-centred symmetric functions (ACSFs).^33^ ACSFs provide a “fingerprint” or “descriptor”, consisting of outputs from multiple two- and three-body functions, effectively encoding the structural features of the nanoclusters. Additional data provided to the agent includes energies and forces, enabling it to learn the impact of configuration changes on these properties. We also incorporated binary vectors to represent various observations, such as whether the agent has encountered an overlapped configuration, dissociated configuration, a local minimum, and the attainment of lower or higher energy minima. Another binary vector indicates the fulfillment of convergence criteria, set to trigger episode termination upon locating five distinct local minimum configurations. These state observations are processed through separate multilayer perceptrons, combined into a state embedding, and subsequently inputted into the critic network.

#### 2.1.2 Action Space Design

The action space (A) consists of all potential actions executable by the agent within the environment. For this study, we devised a discrete action space encompassing two actions, .

- The first action  involves randomly choosing a cluster atom from the total *N* atoms.
- The second action  selects a movement distance of either +2.0 Å or −2.0 Å.

Figure 1b illustrates the action step in nanocluster configuration. In each iteration of the process, the agent employs a stochastic approach to select an atom, as dictated by the initial action, . This atom is then repositioned toward a novel location, the direction of which is determined by a vector originating from the atom’s initial position and pointing toward the nanocluster’s center of mass. The magnitude of this vector is determined by a secondary action, , which specifies the distance the atom is to be moved. This procedural stratagem is pivotal in enabling the agent to traverse various local minima by systematically exploring an array of nanocluster configurations.

#### 2.1.3 Reward Function and Episode Termination

In the development of the DRL framework for exploring nanocluster configurations, we have meticulously crafted the reward function to guide the agent toward identifying a multitude of local energy minima. The reward structure is detailed as follows:

- Penalty for dissociation: actions by the agent that result in dissociation from the cluster are considered as an detrimental outcome. To discourage such events, a negative reward of −10 is implemented, serving as a substantial deterrent against these undesirable configurations.
- Penalty for atom overlap: if the agent’s actions result in overlapping atoms, this outcome is similarly viewed as detrimental. To prevent such events, a negative reward of −10 is applied, serving as a strong deterrent against these unfavorable configurations.
- Penalty for revisiting previous minima: in instances where the agent’s movement results in a local relaxation that does not culminate in overlapping or dissociation, yet converges to a previously identified minimum in the same episode, a similar penalty is applied. To prevent the agent from repetitively exploring already known minima, a penalty is imposed in such cases, mirrored by the same negative reward of −10.
- Reward for discovering new lower energy minimum: the crux of the reward function lies in encouraging the discovery of new local minima. If the agent’s actions lead to the identification of an new local minimum with lower energy value compared to the energy value of the initial configuration, a positive reward is granted. The amount of this reward depends on the difference in energy levels between the newly discovered minimum and the initial configur
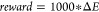
1where Δ*E* is the relative energy with respect to the initial configuration in eV. Specifically, the reward is greater if the new minimum has a lower energy level, thus incentivizing the discovery of energetically favorable configurations.
- Reward for discovering new higher energy minimum: if the agent identifies a new configuration that has a higher energy value than the initial setup, it will receive no reward (i.e., reward = 0). This discourages the exploration of less favorable energy states.

This reward structure is carefully designed to balance between penalizing unproductive explorations and rewarding the discovery of new, energetically favorable configurations, thus guiding the DRL agent in the efficient exploration of nanocluster configuration space.

#### 2.1.4 Termination Criteria

The exploration cycle within an episode concludes under two specific conditions. The primary termination criterion is achieved when the agent successfully identifies five new lower energy minima relative to the initial configuration. This condition marks the completion of a significant learning phase in the exploration of nanocluster configurations. Alternatively, the episode also concludes if the predefined maximum number of steps is reached, ensuring a bound on the exploration time.

### 2.2 Generation of Initial Random Configurations

In our exploration of nanocluster geometries using the DRL framework, generating diverse initial states for each training episode is a crucial aspect. This is achieved either by randomly generating a single geometry or by producing a pair of random geometries through a stochastic process. In the latter case, crossover operations, also known as “mating operations,” are utilized to create a novel hybrid configuration, akin to offspring. These crossover strategies are influenced by the methodologies used in the Birmingham parallel genetic algorithm (BPGA).^34,35^

To broaden the diversity of these initial states and encompass a wider spectrum of potential configurations, we have integrated various mutation operations from the BPGA into the initial random geometry generation phase. This two-step approach, comprising random configuration generation followed by subsequent mutation operations, ensures that the training process begins with a diverse set of initial configurations.

The mutation operations include “move”, “rotate”, “twist”, “partial inversion”, “rattle”, among others, each contributing uniquely to the modification of cluster configurations. We have also include a mutation operation called “do nothing” which is essentially leave the initial cluster configuration intact. Other mutations makes further changes in the initial configuration generated. For example, the skin mutation maintains the stability of the configuration by keeping 80% of the cluster atoms intact while repositioning the remaining 20% randomly around the cluster, thus ensuring both consistency and variability in the structure. The rattle mutation adds dynamism by selecting a random atom and then moving a significant proportion (50% or 75%) of the atoms nearest to this chosen atom, effectively reshaping the cluster’s spatial arrangement. Similarly, the change core mutation alters the very heart of the cluster, fundamentally changing its overall structure. The rotation and twist mutations, by rotating and twisting a portion of the cluster atoms, introduce a complex layer of spatial manipulation into the configuration. The partial inversion operation offers a mirrored alteration by inverting a subgroup of atoms in relation to their geometrical center, adding an element of symmetry to the configuration. Furthermore, the tunnel operation strategically relocates one of the atoms from the furthest point of the center to the opposite side, significantly affecting the spatial dynamics of the cluster. Homotopic mutation is a method specifically developed for bi- or multimetallic nanoclusters, involving the strategic swapping of two different elemental atoms to generate novel configurations.

### 2.3 Identification of Global Minimum

In each episode, the model records all encountered minimum energy configurations and separately stores the lowest energy minimum. Furthermore, a pool of lowest energy configurations is generated, typically comprising a predetermined number (e.g., 10 configurations). Upon locating the specified number of lowest energy configurations, if a new minimum is discovered during the training process, it replaces the highest energy conformation in the existing pool. This iterative process persists until the training reaches its completion. Additionally, at the end of the training steps, the model identifies the GM and other lowest energy configurations from the saved minimum configurations obtained across episodes.

As an alternative approach, after concluding the training steps, a dedicated analysis is undertaken to pinpoint the GM and other configurations with the lowest energy levels. This analytical procedure entails scrutinizing the stored minimum configurations gathered across episodes.

### 2.4 Capabilities of the DRL Framework

As outlined earlier, our DRL framework diligently captures all configurations encountered in each training episode – including minima, overlapped, and dissociated configurations – and aggregates them into trajectories. It specifically logs every minimum energy configuration identified during the training sessions, storing these selectively as trajectories comprised solely of minima. Additionally, it maintains a record of the lowest energy configuration found in each episode separately. These varied trajectory data sets are invaluable for multiple applications, such as developing machine learning force fields and other relevant disciplines. Furthermore, this framework can also function as an automatic generator of diverse nanocluster configurations.

### 2.5 DRL Experiments

In our research, we have experimentally validated the proposed DRL framework across various nanoclusters. For the implementation and testing of our DRL model, we utilized the OpenAI Gym^36^ and Tensorforce^37^ frameworks, both of which are renowned for their robustness and flexibility in handling complex reinforcement learning tasks. Each experimental episode in our DRL framework was configured to run for a maximum of 200 action steps. This constraint ensures a comprehensive exploration of the action space while maintaining computational efficiency. A critical component of our experiments involved the computation of energy and forces for the nanoclusters. For this purpose, we utilized the effective medium theory (EMT) potentials.^38−40^ EMT potentials are well-established in computational materials science for providing reliable and computationally efficient estimations of interatomic forces and energies in metallic systems. By incorporating EMT potentials into our DRL framework, we ensured that the model’s decisions were informed by physically accurate and relevant data, thereby enhancing the reliability of the optimization process. To further enhance the efficiency and scalability of our experiments, we employed multiprocessing parallel environment executions. In this setup, multiple instances of the environment run in parallel, all sharing the same agent and model parameters. This parallelism not only expedites the learning process but also provides a broader and more diverse exploration of the state and action spaces.

In the exploration of optimal nanocluster configurations, the RL agent is tasked to discover five distinct lower energy minima during each episode. The training process is initiated with randomly generated nanocluster configurations as outlined in Section 2.2, which undergo relaxation to local minima. Subsequently, each relaxed configuration is input into the DRL framework for training purposes.

### 2.6 Computational Methods

We employed the effective medium theory (EMT) potentials for the geometry optimization and evaluation of energy and forces in nanoclusters. EMT is a semiempirical interatomic potential widely used in materials science, particularly for studying metallic systems and alloys. Its computational efficiency makes it an ideal choice for modeling extensive atomic systems, especially in scenarios where more computationally intensive methods, such as density functional theory (DFT), are impractical.

EMT is based on the concept that the potential energy of an atom within a material can be effectively represented by an “effective medium” that reflects the influence of the surrounding atomic environment. The total energy of an atom is expressed as a combination of a pairwise interaction term and a contribution from the electron density of neighboring atoms, capturing the essential characteristics of metallic bonding. This approach makes EMT particularly suitable for large-scale simulations of metallic systems where the use of DFT would be computationally prohibitive.

EMT has been extensively applied to a wide range of studies involving metals, alloys, and nanoclusters, including investigations of structural properties, formation energies, surface energies, and defect structures.^21,38−43^ The combination of simplicity and computational efficiency makes EMT a valuable tool in the theoretical investigation of large metallic systems, particularly when more precise quantum mechanical methods are not feasible.

In Section 3, under Section 3.5, we detail the approach employed to identify the global minimum of Ag_48_ using DFT calculations. The Vienna Ab-initio Simulation Package (VASP)^44−46^ was utilized for all DFT computations.

Initially, single-point DFT calculations were performed on all nanocluster configurations identified as unique minima. Subsequently, a subset of low-energy configurations–100 in this specific case–was chosen for full structural relaxation. Both the single-point and relaxation calculations utilized a spin-polarized DFT approach at the gamma-point, employing a plane-wave basis set. The Perdew–Burke–Ernzerhof (PBE)^47^ exchange-correlation functional, within the framework of the generalized gradient approximation (GGA), was used to account for electronic exchange and correlation effects. Ion-electron interactions were represented by the projected augmented wave (PAW) pseudopotentials.^45^ To ensure accurate results, the plane-wave energy cutoff was set to 400 eV, and Methfessel–Paxton^48^ smearing was applied with a sigma value of 0.2 eV to improve convergence. The convergence criteria were set to an energy threshold of 10 eV and a force threshold of 0.01 eV/Å.

## 3 Results and Discussion

In this section, we provide a comprehensive analysis of our DRL experiments conducted on a variety of nanoclusters, encompassing both monometallic and multimetallic types. Our primary focus will be on the monometallic clusters Cu_20_ and Ag_48_, as well as the multimetallic clusters Au_22_Ni_16_ and Cu_4_Pd_5_Ni_6_. These clusters serve as representative examples for our discussion. Detailed results and extensive data from our DRL experiments on other monometallic (Au_44_, Ni_35_, and Pd_27_) and multimetallic alloy nanoclusters (Au_18_Cu_16_, Ag_20_Au_15_, Cu_15_Pd_15_, Cu_22_Ni_20_, Au_12_Pd_15_, Ni_10_Pd_13_) are included in the Supporting Information.

### 3.1 Cu_20_

**Figure 2:**
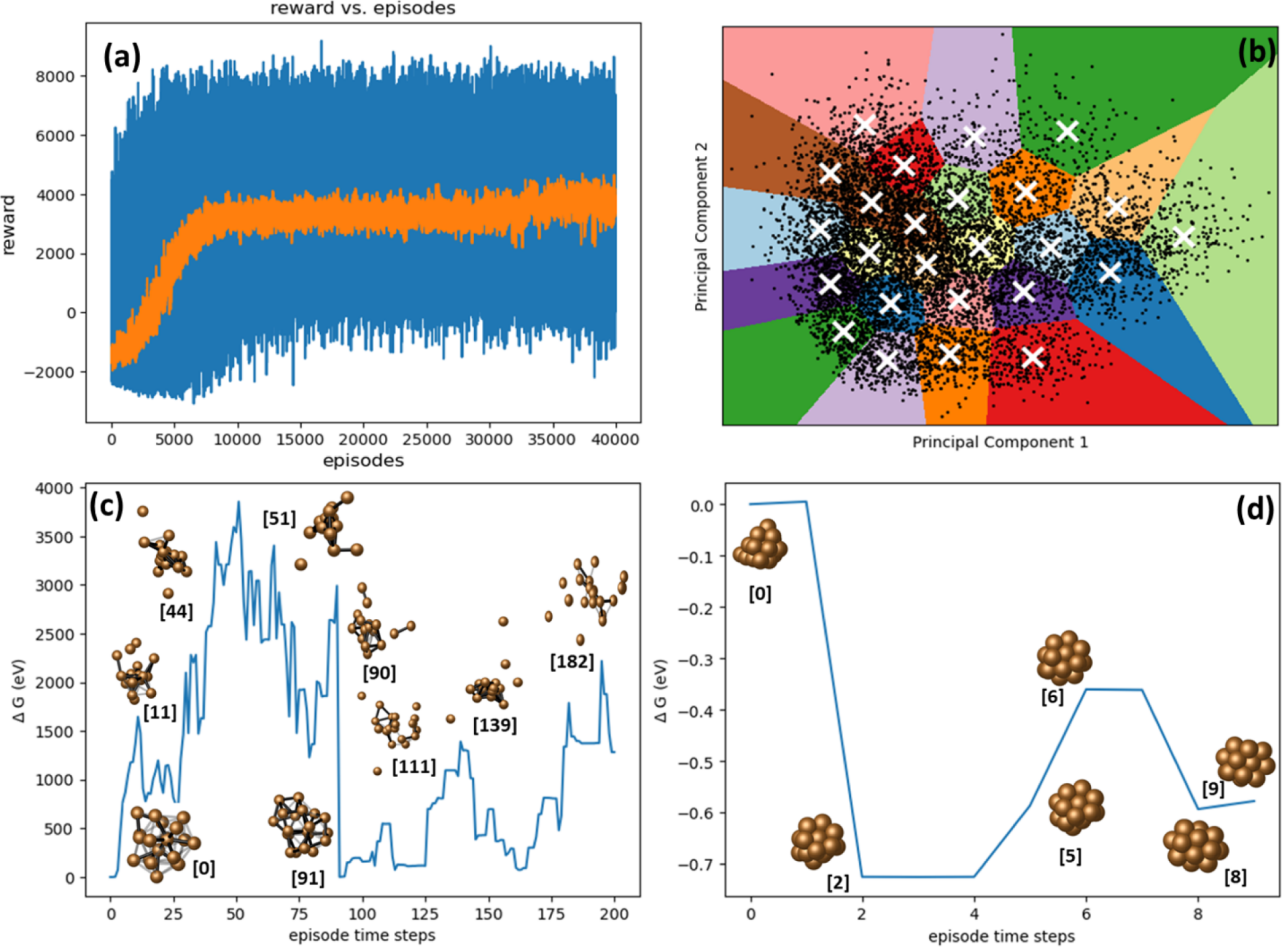
Results of DRL experiments on Cu_20_ nanocluster: (a) progression of episodic rewards throughout the training sessions, represented by blue lines, along with the moving average depicted as an orange curve, highlighting trends over each episode. (b) Application of K-means clustering to analyze unique minimum energy configurations identified during the entire training phase, preceded by a dimensionality reduction process. (c, d) Energy profiles from representative episodes early in the training phase, illustrating the initial model behavior before a stable policy is developed, and later stages after the model has established a learned policy, respectively.

**Figure 3:**
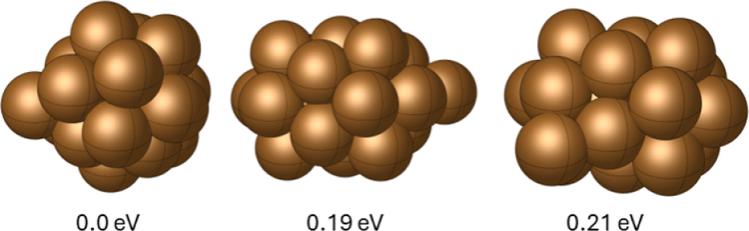
GM and low-energy configurations for Cu_20_.

In Figure 2a, we illustrate the progression of episodic rewards (depicted as blue lines) and the corresponding moving average (shown as a orange curve) for each episode. Figure 3 displays the GM and two lying energy configurations for Cu_20_. The moving average effectively quantifies the average variation in reward values over predetermined time steps, serving as a tool to ascertain trends in the trajectory of rewards. During the initial training phase, the agent primarily encounters negative rewards as a result of generating overlapped or dissociated cluster configurations, signifying the absence of a sufficiently learned policy to guide decision-making toward avoiding such penalties. This period is characterized by a significant degree of exploration, where the agent’s policy is relatively unrefined and “noisy.” As training advances, a noticeable shift occurs; the model begins to exhibit learning. Notably, after approximately 10,000 episodes, the agent demonstrates a proficient ability to identify the five lowest energy minima, beginning from a random initial configuration. Subsequently, a period of reward stabilization is observed, aligning with the agent’s acquisition of a stable and effective policy. This stabilization is a critical milestone in the training process, indicating a successful adaptation and learning by the agent within its environment.

**Figure 4:**
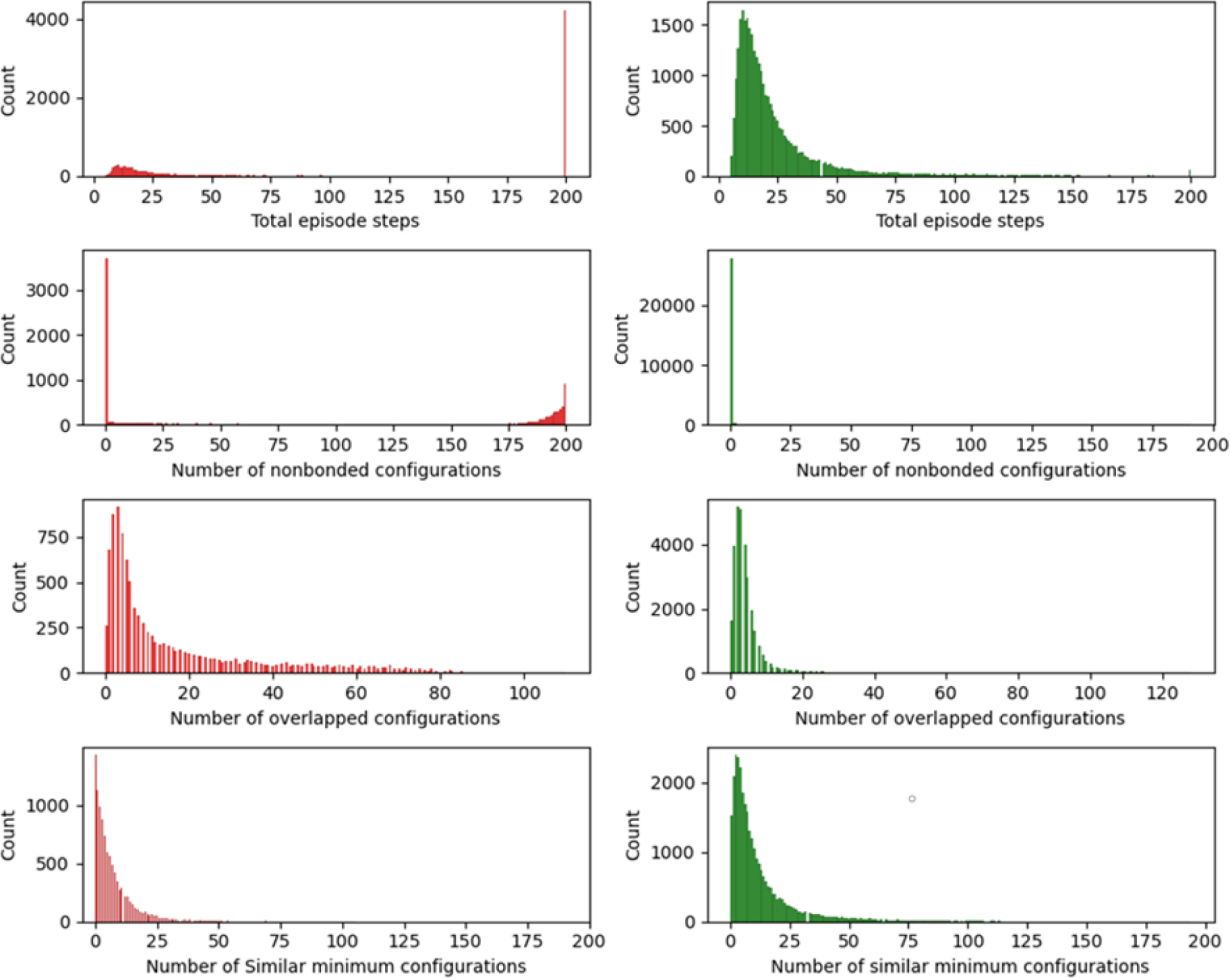
Distribution metrics from DRL experiments on Cu_20_ nanocluster across pre and post policy learning phases: (first row) histograms displaying the total number of steps per episode, contrasting the early training phase (left, red) with the postpolicy learning phase (right, green). Successive rows detail the distributions of nonbonded configurations, overlapped configurations, and similar minimum energy configurations in each episode, showcasing the model’s adaptive behavior and optimization progress through the training periods.

Figure 4 delineates the distribution of the total number of steps per episode, juxtaposing the early training phase against the postpolicy learning phase (represented by the left and right columns, respectively). It is discernible from the histograms that at the onset of training, episode termination is predominantly dictated by the completion of the allotted 200 training steps, reflecting the agent’s inability to locate the required five distinct minima within the specified time frame. Only a negligible fraction of training episodes conclude prior to this limit. However, postlearning a stable policy – after approximately 10,000 episodes – the training episodes terminate well before the maximum threshold of 200 steps. Notably, most episodes conclude within 25 steps after successfully identifying the five lowest energy configurations.

Furthermore, the initial training episodes exhibit a pronounced frequency of more than 175 nonbonded configurations, particularly noticeable in episodes culminating with 200 nonbonded configurations. This observation suggests that the early training predominantly generates nonbonded configurations, which are characterized by fragmented clusters. After the model acquires an efficacious policy, there is a precipitous decline in the number of nonbonded configurations, virtually diminishing to zero. Analogously, the occurrence of overlapping configurations is markedly higher during the initial training phase but substantially decreases following approximately 10,000 training episodes, indicating the model’s enhanced proficiency in generating viable nanocluster configurations and avoiding suboptimal ones as the training progresses.

The distribution plots regarding the number of similar minimum configurations encountered in each episode demonstrate a less pronounced difference between pre- and postpolicy learning training episodes. It is important to recognize that in the early stages of training, due to the prevalence of nonbonded and overlapped configurations, fewer relaxations to local minima occur, thereby precluding a similarity check for minimum configurations. Upon the establishment of an effective policy, episodes conclude expeditiously, and the instances of encountering previously identified similar configurations within an episode are significantly diminished.

These observations collectively highlight the DRL framework’s efficacy in refining the search strategy for optimal nanocluster configurations, with a notable transition from a trial-and-error approach in the early stages to a more informed and strategic exploration following the postpolicy learning phase. The framework demonstrates an enhanced ability to avoid unfavorable configurations and efficiently guide the agent toward the discovery of lower energy states after it has learned the stable policy.

Figure 2c depicts an energy profile from a representative episode in the early stages of training, prior to establishing a stable policy for Cu_20_ nanoclusters. In the initial training phases, the configurations are predominantly disassociated or overlapping, and the agent does not execute relaxation on these configurations, resulting in immediate negative feedback. Furthermore, atom movements from these high-energy states tend to yield additional unfavorable configurations, leading to further negative rewards. In the depicted energy profile in Figure 2c, the episode terminates after 200 time steps without achieving the goal of locating five distinct lower energy minima. However, after completing around 10,000 episodes, the model started learning a policy and the reward is stabilized.

Conversely, Figure 2d illustrates the configurations and energies of nanoclusters found after the agent has learned a stable policy. In this representative episode, the agent successfully terminates the episode in just 10 time steps, identifying five unique minima and four similar minima. This outcome demonstrates the agent’s acquisition of a policy enabling wise movements to avoid generating overlapped or dissociated configurations.

Despite the primary termination criterion being the discovery of five distinct local minima in each episode, the agent actively explores a greater number of higher energy configurations, thereby enabling the exploration of a more diverse configuration space by relocating atoms from these high-energy states.

Subsequently, we aggregated all the minimal configurations generated throughout the training phase, conducting a thorough analysis to identify distinct configurations. Figure 2b illustrates the use of K-means clustering on these unique minimum energy configurations, following a process of dimensionality reduction. This reduction involved mapping the data from its original high-dimensional space to a two-dimensional plane using principal component analysis (PCA). This transformation enabled us to visualize the data and its respective clusters within this reduced dimensional space. The primary purpose of employing PCA was to effectively decrease the data set’s dimensionality, preserving as much of the original data’s variance as possible. The amount of variance captured in each principal component is pivotal, as it reflects the extent to which that component contains information from the original data set.

In our analysis, as depicted in the plot, we have identified 25 distinct clusters through the application of elbow analysis. It is important to note that the dimensionality reduction achieved by PCA, utilizing only two principal components, accounts for only 19% of the total variance in the data. This relatively low percentage indicates that the majority of the data’s variability is not captured in the two-dimensional representation. As such, while the two principal components may capture the most significant patterns or trends, they may miss more subtle nuances present in the original high-dimensional space. Clustering in the reduced two-dimensional space can reveal certain structures or groupings that are prominent with respect to the principal components. However, due to the low variance retained (19%), these clusters might not fully represent the true, complex relationships in the original high-dimensional data.

The K-means clustering plots illustrate that within each cluster region, the data points corresponding to minimum energy configurations exhibit broad dispersion. This dispersion highlights the diversity of the minimum energy configurations within each cluster, a significant observation considering the limited variance (19%) captured by the two principal components. Given the extensive dispersion observed among the data points within each cluster, there is a potential consideration for further subdividing these clusters into smaller groups. Such a subdivision might provide a more granular view of the data, potentially revealing more intricate patterns or substructures within each cluster.

### 3.2 Ag_48_

**Figure 5:**
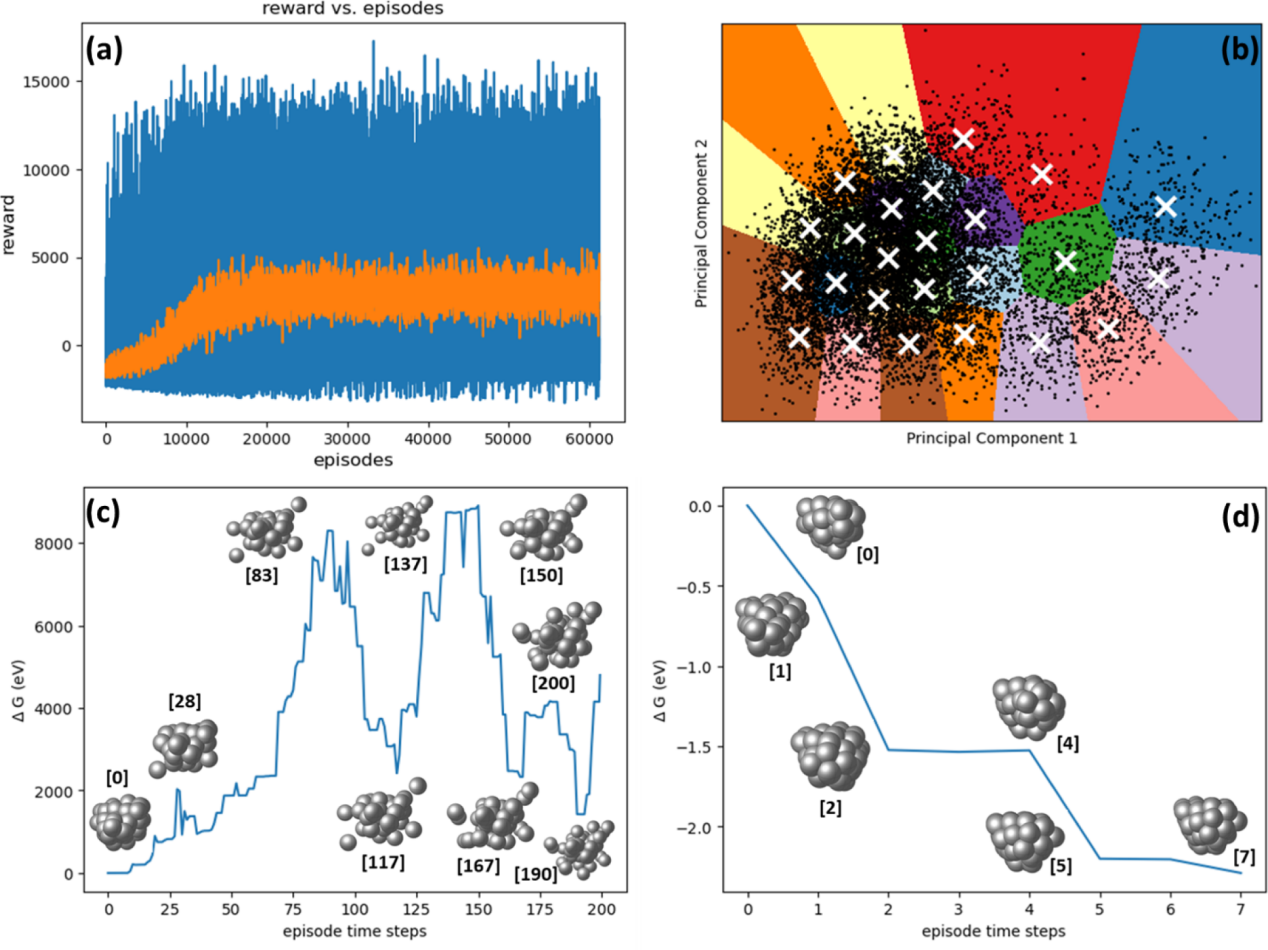
Results of DRL experiments on Ag_48_ nanocluster. (a) Progression of episodic rewards throughout the training sessions, represented by blue lines, along with the moving average depicted as an orange curve, highlighting trends over each episode. (b) Application of K-means clustering to analyze unique minimum energy configurations identified during the entire training phase, preceded by a dimensionality reduction process. (c, d) Energy profiles from representative episodes early in the training phase, illustrating the initial model behavior before a stable policy is developed, and later stages after the model has established a learned policy, respectively.

**Figure 6:**
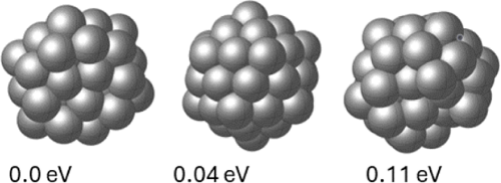
GM and low-energy configurations for Ag_48_.

**Figure 7:**
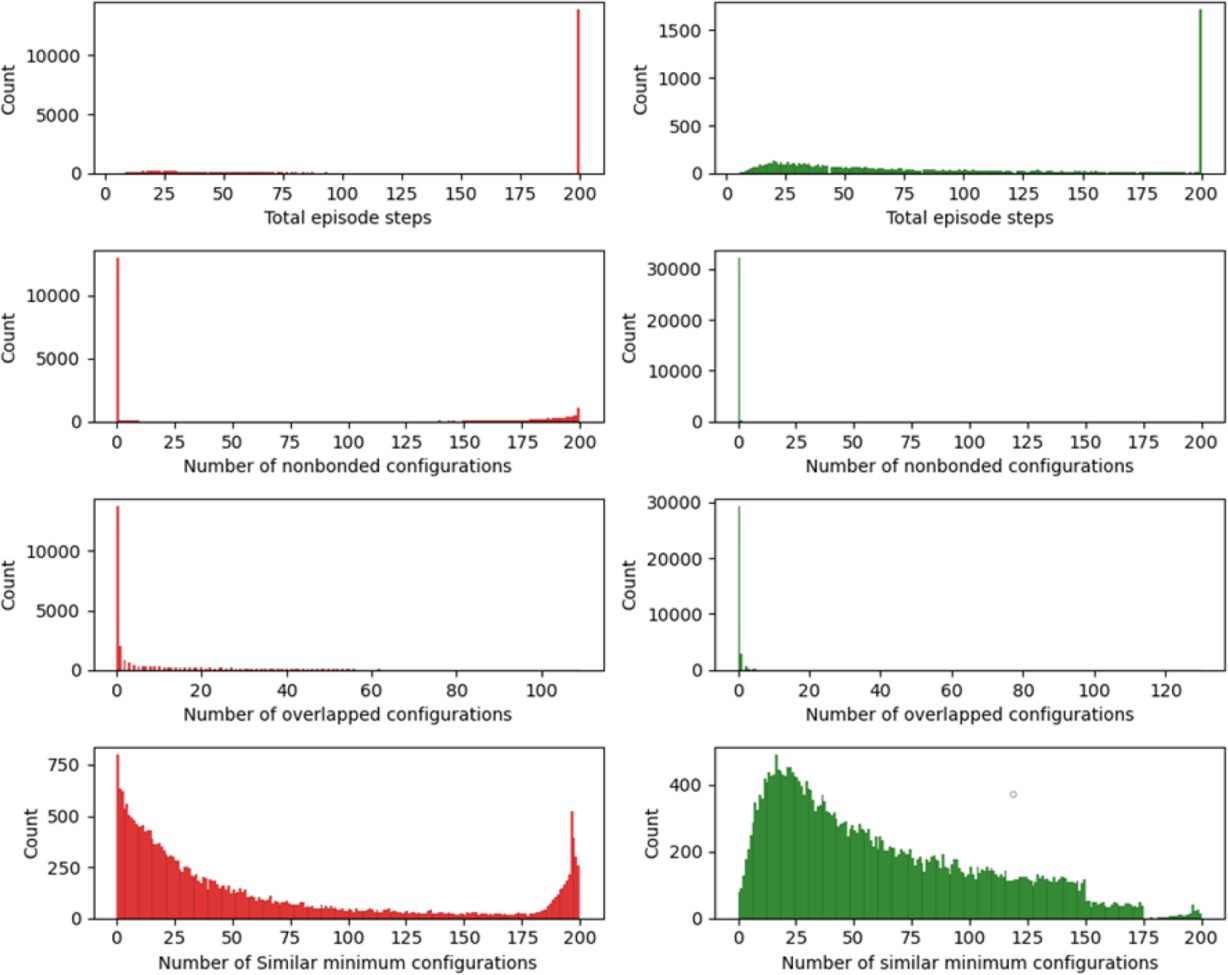
Distribution metrics from DRL experiments on Ag_48_ nanocluster across pre and post policy learning phases: (first row) histograms displaying the total number of steps per episode, contrasting the early training phase (left, red) with the postpolicy learning phase (right, green). Successive rows detail the distributions of nonbonded configurations, overlapped configurations, and similar minimum energy configurations in each episode, showcasing the model’s adaptive behavior and optimization progress through the training periods.

We extend the application of our DRL framework to larger nanoclusters, using Ag_48_ as a representative example. The results of this extension are illustrated in Figures 5–7. These findings exhibit a notable similarity to the observations recorded for the Cu_20_ nanocluster, suggesting a consistent effectiveness of the DRL framework across different sizes and compositions of nanoclusters. Analysis of the reward trajectories indicates that the model achieves a stable policy after approximately 15,000 training episodes. This extended training duration, when compared to the smaller Cu_20_ nanocluster, can be attributed to the increased complexity of the larger Ag_48_ nanocluster. Furthermore, the necessity for the model to select one cluster atom from 48 available cluster atoms significantly escalates the dimensionality of the DRL problem. Initially, the training episodes often concluded without identifying the five lowest energy states, reaching the maximum limit of 200 steps. However, once a stable policy was established, the model consistently identified lower energy configurations within significantly fewer steps, frequently well before reaching the imposed step limit.

In alignment with observations from the Cu_20_ cluster, the frequency distributions of nonbonded and overlapped configurations during the training episodes initially exhibit a propensity for the model to generate a considerable number of nonbonded configurations, particularly peaking at 200 configurations. This trend signifies a predominance of nonbonded configurations in the initial training phase as we have observed in the case of Cu_20_. Additionally, the model initially presented an elevated count of overlapped nanocluster configurations, indicative of the learning curve before achieving a stable policy. Postpolicy stabilization, the incidences of both nonbonded and overlapped configurations diminished markedly, virtually to nil. This suggests that the model’s policy learned to avoid these suboptimal configurations as training progressed.

Figure 5c,d displays the structural configurations and associated energy levels of nanoclusters in two representative episodes, both prior to and subsequent to the agent’s mastery of a stable policy. These findings align closely with those observed in the previously discussed Cu_20_ example. During the initial stages of training, the model concluded its training process upon reaching the preset number of training steps, failing to identify the five distinct lower energy minima, as showcased in Figure 5c. In contrast, Figure 5d illustrates that, mirroring the Cu_20_ cluster outcomes, the agent eventually terminated episodes more efficiently, successfully pinpointing five unique lower energy minima. This progression underscores the agent’s evolved strategy, characterized by judicious movements that effectively circumvent the creation of overlapping or disjointed configurations. Upon the assimilation of this policy, the model’s reward metrics exhibited a consistent elevation to higher levels.

Additionally, the Supporting Information includes an examination of our DRL framework applied to larger nanoclusters Au_44_ and Ni_35_ nanoclusters and a medium sized nanocluster Pd_27_. The resulting performance metrics and observations for these clusters mirror those recorded for these nanoclusters, demonstrating the robustness of our model across different cluster sizes and compositions. To avoid redundancy, we have elected not to reiterate these findings in the main text.

### 3.3 Au_22_Ni_16_

**Figure 8:**
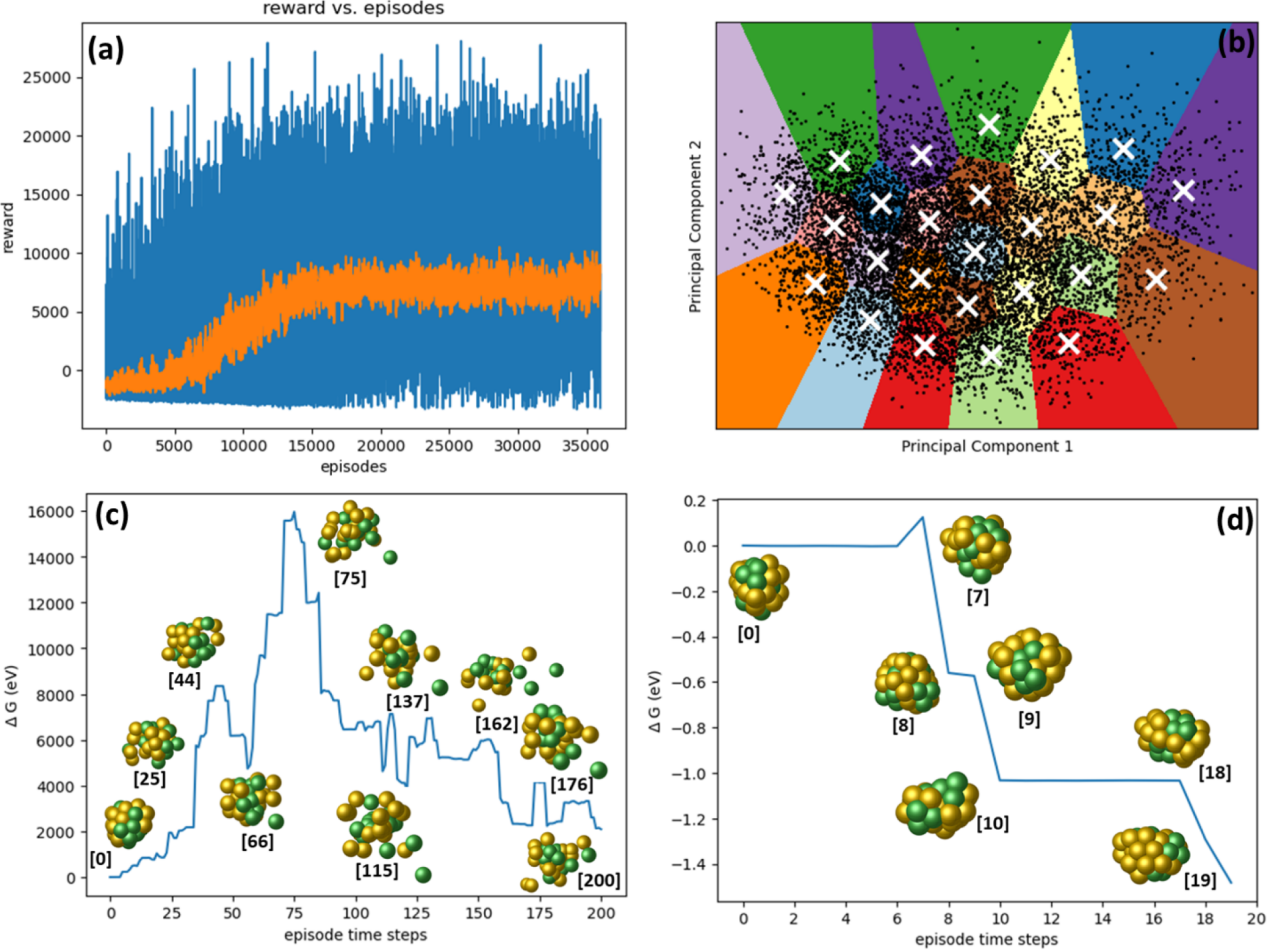
Results of DRL experiments on Au_22_Ni_16_ nanocluster: (a) progression of episodic rewards throughout the training sessions, represented by blue lines, along with the moving average depicted as an orange curve, highlighting trends over each episode. (b) Application of K-means clustering to analyze unique minimum energy configurations identified during the entire training phase, preceded by a dimensionality reduction process. (c, d) Energy profiles from representative episodes early in the training phase, illustrating the initial model behavior before a stable policy is developed, and later stages after the model has established a learned policy, respectively.

**Figure 9:**
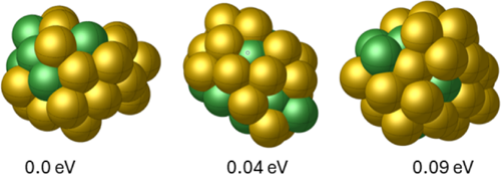
GM and low-energy configurations for Au_22_Ni_16_.

**Figure 10:**
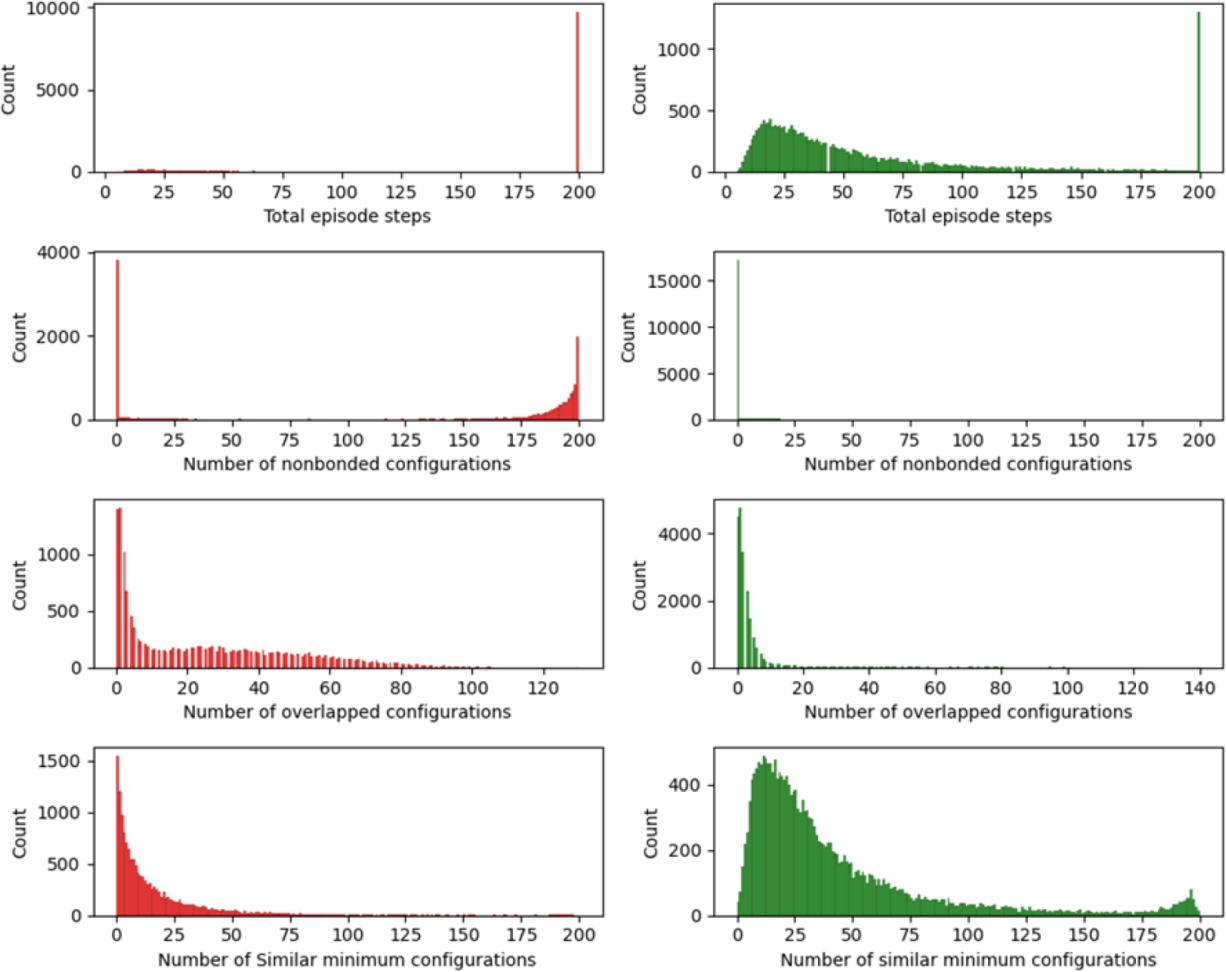
Distribution metrics from DRL experiments on Au_22_Ni_16_ nanocluster across pre and post policy learning phases: (first row) histograms displaying the total number of steps per episode, contrasting the early training phase (left, red) with the postpolicy learning phase (right, green). Successive rows detail the distributions of nonbonded configurations, overlapped configurations, and similar minimum energy configurations in each episode, showcasing the model’s adaptive behavior and optimization progress through the training periods.

We further extend the demonstration of the DRL model’s capabilities by presenting the outcomes for bimetallic Au_22_Ni_16_ nanoalloy clusters in Figures 8–10. Distinct from the monometallic cases previously discussed, the bimetallic nanoclusters possess a fingerprint feature representation that is twice as large. For example, the feature vector for Au_22_Ni_16_ is 1520 in length, whereas for monometallic clusters such as Ni_35_ and Au_44_, the lengths are 665 and 836, respectively. Despite anticipations of extended training durations due to the increased dimensionality and feature vector size associated with bimetallic nanoclusters, the model generally achieved a stable policy within approximately 20,000 training episodes. Given the similarity of these results to those observed in the monometallic cluster cases, we have chosen not to elaborate on these findings, which largely echo the patterns and conclusions already discussed. Additional examples of the experiment’s performance on bimetallic nanoclusters are available in the Supporting Information.

### 3.4 Multimetallic System: Cu_4_Pd_5_Ni_6_

**Figure 11:**
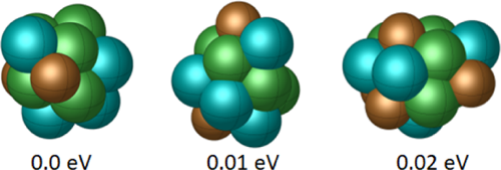
GM and low-energy configurations for Cu_4_Pd_5_Ni_6_.

In our conclusive demonstration of the DRL model’s capabilities for a multimetallic system containing more than two metal atoms, we conducted studies on Cu_4_Pd_5_Ni_6_ using our DRL approach. Figure 11 presents the global minimum configuration, along with two other low-lying energy configurations. As the results are consistent with observations from other examples, we provide the detailed results in the Supporting Information without reiterating the same findings described earlier.

When dealing with multimetallic systems, the DRL framework encounters significant challenges due to the curse of dimensionality. As more metallic components are added, the dimensionality of the problem increases, leading to a substantial expansion in the feature vector size – potentially doubling for bimetallic systems and increasing even more for multimetallic systems. This larger feature vector makes the optimization landscape more complex, with a more rugged potential energy surface and multiple local minima, complicating the search for the global minimum. The DRL framework must adapt its learning policy to navigate this high-dimensional space effectively, which can slow down the training process and require more computational resources.

Moreover, the variability in atomic sizes, electronegativities, and bonding characteristics among different metals adds another layer of difficulty. These factors make it harder to accurately predict the energetics and geometry of multimetallic nanoclusters, complicating the reward function used in the DRL framework. As a result, while the framework is versatile, applying it to multimetallic systems is inherently more challenging than to monometallic or bimetallic systems due to the increased complexity and the difficulties in efficiently searching for global minima in a high-dimensional optimization landscape with an expanded feature vector size.

### 3.5 Strategies for Global Minimum Search at the DFT Level

**Figure 12:**
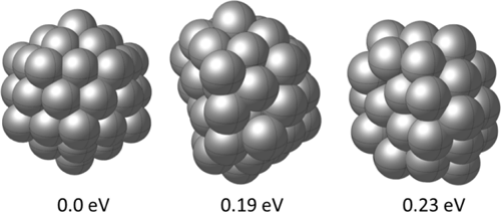
GM and two-lying configurations of Ag_48_ evaluated at the DFT level.

Due to the computational impracticality of performing density functional theory (DFT) level relaxation on all nanoclusters, we adopted an alternative strategy for locating the GM at the DFT level. Our DRL framework captures all minima encountered in each episode. From these recorded minima, we first identify the unique configurations and then perform single point energy calculations at the DFT level. Based on these energy values, we then select a predetermined number of the lowest energy configurations for further relaxation at the DFT level. Our methodology is based on the assumption that initiating relaxation from a local point on the PES is likely to lead to the nearest local minimum. By sampling the lowest energy configurations among the complete set of distinct configurations, we are confident in our approach’s ability to locate the GM configuration on the PES. As an example, Figure 12 shows the DFT level GM found for Ag_48_ adopting the above strategy. The GM configuration identified using DFT differs from the GM generated by the DRL framework because the latter was based on EMT potentials. The DRL framework utilizes EMT potentials to efficiently explore and optimize the nanocluster configurations, which provides a computationally feasible approach for scanning a large number of potential geometries. However, when these EMT-optimized configurations are further refined using DFT, the resulting GM may differ due to the higher accuracy and different underlying physical principles of DFT compared to EMT. This discrepancy is expected as DFT provides a more precise evaluation of interatomic interactions, leading to potentially different optimal configurations than those predicted by the semiempirical EMT approach. For broader applicability, researchers may choose any lower level of theory for this purpose or even develop a machine learning-based force field to sample configurations using the DRL framework.

## 4 Conclusions

We have designed and developed a novel deep reinforcement learning (DRL) framework and highlighted the versatility and precision of DRL in identifying optimal configurations of pure nanoclusters and alloyed nanoclusters. It demonstrates DRL’s capacity to handle diverse nanocluster types, including both mono- and multimetallic compositions, and efficiently navigate their complex potential energy landscapes. Notably, DRL successfully identifies low-energy configurations even in the face of increasing cluster size and feature vector dimensions. Key findings include DRL’s adaptability to complex energy landscapes and its proficiency in navigating high-dimensional spaces.

In its initial training phases, the model, beginning from a random geometric configuration, tends to produce configurations that often result in nonbonded, dissociated fragments, or overlapping structures. However, as it progresses and learns a stable policy, the model adapts to avoid creating such nonbonded and overlapping configurations, opting instead for more strategic and effective movements. This evolution in the model’s approach indicates a significant improvement in its capability to discern and generate more viable nanocluster configurations. As a result, the model efficiently locates five distinct local minimum energy configurations in a significantly reduced number of time steps, demonstrating its effectiveness in identifying optimal structures quickly. Starting from random nanocluster configurations in each training episode, the model is tasked with finding five lower energy configurations. Over a sufficiently large number of episodes, this approach leads to the exploration of a broader range of local minima on the PES. Typically, in most training episodes, the model not only finds lower energy configurations but also identifies a number of higher energy minimum configurations. This process enhances the model’s ability to locate a more diverse array of minima in each episode.

Our DRL framework effectively captures and catalogs every encountered configuration throughout training episodes, including minima, overlapped, and dissociated states, compiling these into detailed trajectories. It specifically logs each minimum energy configuration, organizing these select instances into distinct trajectories composed entirely of energy minima. Additionally, the framework separately maintains records of the lowest energy configuration identified in each training episode. These comprehensive trajectory data sets prove essential for various scientific pursuits, such as the creation of machine learning-driven force fields and beyond. Furthermore, the framework’s capability to autonomously generate diverse nanocluster configurations underscores the potential of DRL as a robust tool in materials science, particularly in the optimization and discovery of novel nanomaterials.

## Data Availability

The code used in this work is available at https://github.com/rajeshkochi444/clusgm_drl. The software is open-sourced and licensed under MIT License.

## Supporting Information Available

Data from our DRL experiments on monomeallic nanoclusters: Au_44_, Ni_35_, and Pd_27_; alloy nanoclusters: Au_18_Cu_16_, Ag_20_Au_1_5, Cu_15_Pd_15_, Cu_22_Ni_20_, Au_12_Pd_15_, Ni_10_Pd_13_, and Cu_4_Pd_5_Ni_6_; hyperparameter settings for DRL experiments (PDF)

## References

[ref1] Olveira S.; Forster S. P.; Seeger S. Nanocatalysis: Academic discipline and industrial realities. J. Nanotechnol. 2014, 2014, 324089 10.1155/2014/324089.

[ref2] Somwanshi S. B.; Somvanshi S. B.; Kharat P. B. Nanocatalyst: A Brief Review on Synthesis to Applications. J. Phys.: Conf. Ser. 2020, 1644, 012046 10.1088/1742-6596/1644/1/012046.

[ref3] Dai Y.; Wang Y.; Liu B.; Yang Y. Metallic nanocatalysis: An accelerating seamless integration with nanotechnology. Small 2015, 11, 268–289. 10.1002/smll.201400847. 25363149

[ref4] Piccolo L. Restructuring effects of the chemical environment in metal nanocatalysis and single-atom catalysis. Catal. Today 2021, 373, 80–97. 10.1016/j.cattod.2020.03.052.

[ref5] Zhai H.; Alexandrova A. N. Fluxionality of Catalytic Clusters: When It Matters and How to Address It. ACS Catal. 2017, 7, 1905–1911. 10.1021/acscatal.6b03243.

[ref6] Astruc D. Introduction: Nanoparticles in Catalysis. Chem. Rev. 2020, 120, 461–463. 10.1021/acs.chemrev.8b00696. 31964144

[ref7] Ferrando R.; Jellinek J.; Johnston R. L. Nanoalloys: From theory to applications of alloy clusters and nanoparticles. Chem. Rev. 2008, 108, 845–910. 10.1021/cr040090g. 18335972

[ref8] Shan S.; Luo J.; Wu J.; Kang N.; Zhao W.; Cronk H.; Zhao Y.; Joseph P.; Petkov V.; Zhong C.-J. Nanoalloy catalysts for electrochemical energy conversion and storage reactions. RSC Adv. 2014, 4, 42654–42669. 10.1039/C4RA05943C.

[ref9] Jellinek J. Nanoalloys: Tuning properties and characteristics through size and composition. Faraday Discuss 2008, 138, 11–35. 10.1039/b800086g. 18447006

[ref10] Johnston R. L. Evolving better nanoparticles: Genetic algorithms for optimizing cluster geometries. J. Chem. Soc., Dalton Trans. 2003, 3, 4193–4207. 10.1039/b305686d.

[ref11] Wales D. J.; Doye J. P. Global optimization by basin-hopping and the lowest energy structures of Lennard-Jones clusters containing up to 110 atoms. J. Phys. Chem. A 1997, 101, 5111–5116. 10.1021/jp970984n.

[ref12] Hohl D.; Jones R. O.; Car R.; Parrinello M. Structure of sulfur clusters using simulated annealing: S2 to S13. J. Chem. Phys. 1988, 89, 6823–6835. 10.1063/1.455356.

[ref13] Zhang J.; Dolg M. ABCluster: The artificial bee colony algorithm for cluster global optimization. Phys. Chem. Chem. Phys. 2015, 17, 24173–24181. 10.1039/C5CP04060D. 26327507

[ref14] Lv J.; Wang Y.; Zhu L.; Ma Y. Particle-swarm structure prediction on clusters. J. Chem. Phys. 2012, 137 (8), 084104 10.1063/1.4746757. 22938215

[ref15] Zhai H.; Alexandrova A. N. Ensemble-Average Representation of Pt Clusters in Conditions of Catalysis Accessed through GPU Accelerated Deep Neural Network Fitting Global Optimization. J. Chem. Theory Comput. 2016, 12, 6213–6226. 10.1021/acs.jctc.6b00994. 27951667

[ref16] Raju R. K.; Sivakumar S.; Wang X.; Ulissi Z. W. Cluster-MLP: An Active Learning Genetic Algorithm Framework for Accelerated Discovery of Global Minimum Configurations of Pure and Alloyed Nanoclusters. J. Chem. Inf. Model. 2023, 63, 6192–6197. 10.1021/acs.jcim.3c01431. 37824704 PMC10598790

[ref17] Wang Y.; Liu S.; Lile P.; Norwood S.; Hernandez A.; Manna S.; Mueller T. Accelerated prediction of atomically precise cluster structures using on-the-fly machine learning. npj Comput. Mater. 2022, 8 (1), 64–66. 10.1038/s41524-022-00856-x.

[ref18] Han S.; Barcaro G.; Fortunelli A. Unfolding the structural stability of nanoalloys via symmetry-constrained genetic algorithm and neural network potential. npj Comput. Mater 2022, 8, 121 10.1038/s41524-022-00807-6.

[ref19] Bisbo M. K.; Hammer B. Global optimization of atomic structure enhanced by machine learning. Phys. Rev. B 2022, 105, 245404 10.1103/PhysRevB.105.245404.

[ref20] Deringer V. L.; Pickard C. J.; Csányi G. Data-Driven Learning of Total and Local Energies in Elemental Boron. Phys. Rev. Lett. 2018, 120, 156001 10.1103/PhysRevLett.120.156001. 29756876

[ref21] Yoon J.; Cao Z.; Raju R. K.; Wang Y.; Burnley R.; Gellman A. J.; Farimani A. B.; Ulissi Z. W. Deep reinforcement learning for predicting kinetic pathways to surface reconstruction in a ternary alloy. Machine Learn.: Sci. Technol. 2021, 2, 045018 10.1088/2632-2153/ac191c.

[ref22] Behler J. Constructing high-dimensional neural network potentials: A tutorial review. Int. J. Quantum Chem. 2015, 115, 1032–1050. 10.1002/qua.24890.

[ref23] Zhou Z.; Kearnes S.; Li L.; Zare R. N.; Riley P. Optimization of Molecules via Deep Reinforcement Learning. Sci. Rep. 2019, 9 (1), 10752 10.1038/s41598-019-47148-x. 31341196 PMC6656766

[ref24] Deringer V. L.; Caro M. A.; Csányi G. Machine Learning Interatomic Potentials as Emerging Tools for Materials Science. Adv. Mater. 2019, 31 (46), 1902765 10.1002/adma.201902765. 31486179

[ref25] Schütt K. T.; Kessel P.; Gastegger M.; Nicoli K. A.; Tkatchenko A.; Müller K.-R. SchNetPack: A Deep Learning Toolbox For Atomistic Systems. J. Chem. Theory Comput. 2019, 15, 448–455. 10.1021/acs.jctc.8b00908. 30481453

[ref26] Zhou Z.; Li X.; Zare R. N. Optimizing Chemical Reactions with Deep Reinforcement Learning. ACS Cent. Sci. 2017, 3, 1337–1344. 10.1021/acscentsci.7b00492. 29296675 PMC5746857

[ref27] Schütt K. T.; Kindermans P.-J.; Sauceda H. E.; Chmiela S.; Tkatchenko A.; Müller K.-R. SchNet: a continuous-filter convolutional neural network for modeling quantum interactions. In Proceedings of the 31st International Conference on Neural Information Processing Systems Red Hook, NY, USA; NIPS, 2017; pp. 992 1002.

[ref28] Sutton R. S.; Barto A. G. Reinforcement learning: an introduction; MIT press, 2018.

[ref29] Schulman J.; Levine S.; Abbeel P.; Jordan M.; Moritz P. Trust Region Policy Optimization. In Proceedings of the 32nd International Conference on Machine Learning Lille, France; JMLR, 2015; pp. 1889 1897.

[ref30] Sutton R. S.; McAllester D.; Singh S.; Mansour Y. Policy Gradient Methods for Reinforcement Learning with Function Approximation. Advances in Neural Information Processing Systems; MIT Press, 1999, 1057–1063

[ref31] Mnih V.; Badia A. P.; Mirza M.; Graves A.; Lillicrap T.; Harley T.; Silver D.; Kavukcuoglu K. Asynchronous Methods for Deep Reinforcement Learning. In Proceedings of The 33rd International Conference on Machine Learning New York, New York, USA; PMLR, 2016; pp. 1928 1937.

[ref32] Haarnoja T.; Zhou A.; Hartikainen K.; Tucker G.; Ha S.; Tan J.; Kumar V.; Zhu H.; Gupta A.; Abbeel P., Soft Actor-Critic Algorithms and Applications; arXiv, 2018. preprint arXiv:1812.05905.

[ref33] Behler J.; Parrinello M. Generalized Neural-Network Representation of High-Dimensional Potential-Energy Surfaces. Phys. Rev. Lett. 2007, 98, 146401 10.1103/PhysRevLett.98.146401. 17501293

[ref34] Davis J. B.; Shayeghi A.; Horswell S. L.; Johnston R. L. The Birmingham parallel genetic algorithm and its application to the direct DFT global optimization of IrN (N = 10–20) clusters. Nanoscale 2015, 7, 14032–14038. 10.1039/C5NR03774C. 26239404

[ref35] Jäger M.; Schäfer R.; Johnston R. L. GIGA: A versatile genetic algorithm for free and supported clusters and nanoparticles in the presence of ligands. Nanoscale 2019, 11, 9042–9052. 10.1039/C9NR02031D. 31025685

[ref36] Brockman G.; Cheung V.; Pettersson L.; Schneider J.; Schulman J.; Tang J.; Zaremba W. Openai gym; arXiv, 2016, 1–4

[ref37] Kuhnle A.; Schaarschmidt M.; Fricke K. Tensorforce: a TensorFlow Library For Applied Reinforcement Learning, 2017. https://github.com/tensorforce/tensorforce (accessed January 01, 2023).

[ref38] Tadmor E. B.; Elliott R. S.; Sethna J. P.; Miller R. E.; Becker C. A. The potential of atomistic simulations and the knowledgebase of interatomic models. JOM 2011, 63, 17–17. 10.1007/s11837-011-0102-6.

[ref39] Jacobsen K. W.; Norskov J. K.; Puska M. J. Interatomic interactions in the effective-medium theory. Phys. Rev. B 1987, 35, 7423–7442. 10.1103/PhysRevB.35.7423. 9941045

[ref40] Jacobsen K. W.; Stoltze P.; Nørskov J. K. A semi-empirical effective medium theory for metals and alloys. Surf. Sci. 1996, 366 (2), 394–402. 10.1016/0039-6028(96)00816-3.

[ref41] Bailey N. P.; Schiøtz J.; Jacobsen K. W. Simulation of Cu-Mg metallic glass: Thermodynamics and structure. Phys. Rev. B 2004, 69, 144205 10.1103/PhysRevB.69.144205.

[ref42] Paduraru A.; Kenoufi A.; Bailey N.; Schiøtz J. An Interatomic Potential for Studying CuZr Bulk Metallic Glasses. Adv. Eng. Mater. 2007, 9, 505–508. 10.1002/adem.200700047.

[ref43] Gavnholt J.; Schiøtz J. Structure and reactivity of ruthenium nanoparticles. Phys. Rev. B 2008, 77, 035404 10.1103/PhysRevB.77.035404.

[ref44] Kresse G.; Furthmüller J. Efficient iterative schemes for ab initio total-energy calculations using a plane-wave basis set. Phys. Rev. B 1996, 54, 11169–11186. 10.1103/PhysRevB.54.11169. 9984901

[ref45] Kresse G.; Joubert D. From ultrasoft pseudopotentials to the projector augmented-wave method. Phys. Rev. B 1999, 59, 1758–1775. 10.1103/PhysRevB.59.1758.

[ref46] Kresse G.; Hafner J. Ab initio molecular dynamics for liquid metals. Phys. Rev. B 1993, 47, 558–561. 10.1103/PhysRevB.47.558. 10004490

[ref47] Perdew J. P.; Burke K.; Ernzerhof M. Generalized Gradient Approximation Made Simple. Phys. Rev. Lett. 1996, 77, 3865–3868. 10.1103/PhysRevLett.77.3865. 10062328

[ref48] Methfessel M.; Paxton A. T. High-precision sampling for Brillouin-zone integration in metals. Phys. Rev. B 1989, 40, 3616–3621. 10.1103/PhysRevB.40.3616. 9992329

