# Supplementary material for: Exploring Nanocluster Potential Energy Surfaces via Deep Reinforcement Learning: Strategies for Global Minimum Search
Source: J Phys Chem A. 2024 Oct 14;128(42):9122–34. doi: 10.1021/acs.jpca.4c04416 (PMC11514025; doi:10.1021/acs.jpca.4c04416)
Supplement: Supplementary file 1 — jp4c04416_si_001.pdf [file jp4c04416_si_001.pdf]

Supporting Information

Exploring Nanocluster Potential Energy Surfaces via Deep Reinforcement Learning: Strategies for Global Minimum Search

Rajesh K. Raju, [1,2]

[1)]National Research Council Canada, Clean Energy Innovation (CEI) Research Centre, Mississauga, Ontario L5K 1B4

[2)]School of Chemistry, University of Birmingham, Birmingham B15 2TT, U.K

Table S1: Hyperparameter settings used for DRL training

agent	TRPO
batch size	10
learning_rate	1e-2,
memory	40000,
max_episode_timesteps	200
parallel_interactions	Number of processes
Exploration	type='decaying', unit='timesteps', decay='exponential', initial_value=0.3, decay_steps=1000, d ecay_rate=0.5

Results of DRL Experiments on:

Monometallic Clusters: Au_{44}, Ni_{35}, Pd_{27}

Bimetallic Clusters: $Au_{18}Cu_{16}$, $Ag_{20}Au_{15}$, $Cu_{15}Pd_{15}$, $Cu_{22}Ni_{20}$, $Au_{12}Pd_{15}$, $Ni_{10}Pd_{13}$

Trimetallic Clusters: $Cu_4Pd_5Ni_6$

For each nanocluster, we have provided:

- Progression of episodic rewards throughout the training sessions, represented by blue lines, along with the moving average depicted as an orange curve, highlighting trends over each episode.
- pplication of K-means clustering to analyze unique minimum energy configurations identified during the entire training phase, preceded by a dimensionality reduction process.
- Energy profiles from representative episodes early in the training phase, illustrating the initial model behavior before a stable policy is developed, and later stages after the model has established a learned policy, respectively.
- Distribution Metrics from DRL Experiments on Ag_{48} Nanocluster Across Pre and Post Policy Learning Phases: (First Row) Histograms displaying the total number of steps per episode, contrasting the early training phase (left, red) with the post-policy learning phase (right, green).
- Successive rows detail the distributions of non-bonded configurations, overlapped configurations, and similar minimum energy configurations in each episode, showcasing the model's adaptive behavior and optimization progress through the training periods.

1. Au_{44}

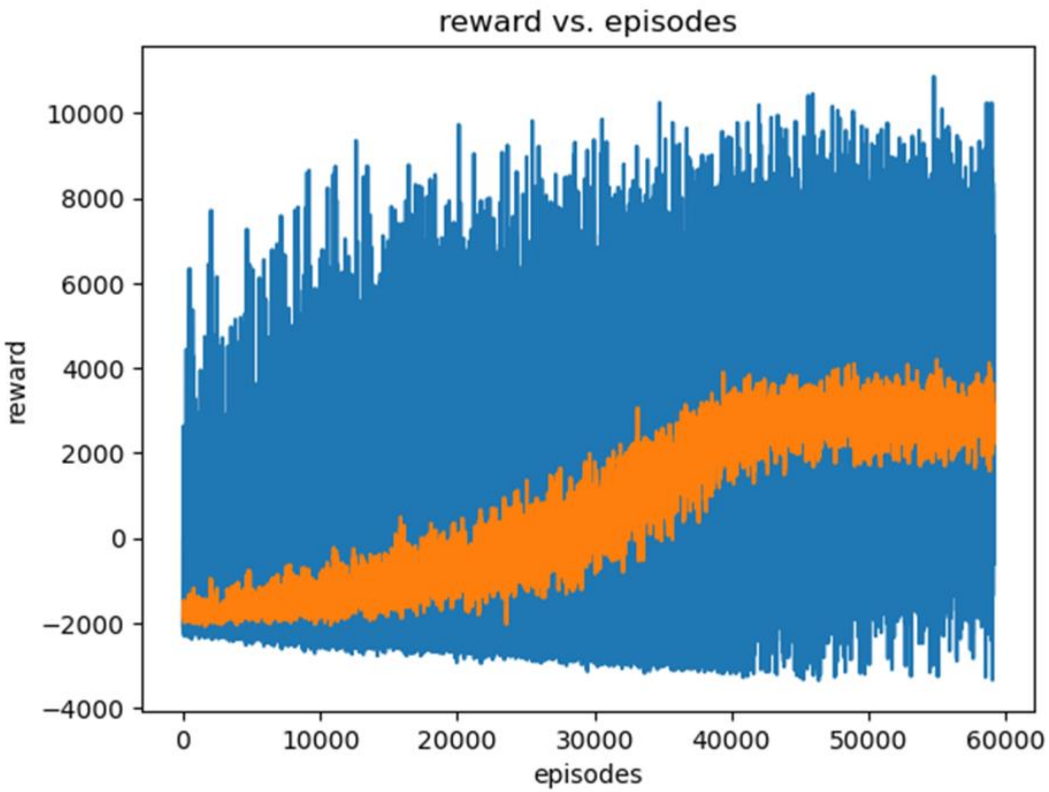
reward vs. episodes
reward
episodes
10000
8000
6000
4000
2000
0
−2000
−4000
0
10000
20000
30000
40000
50000
60000

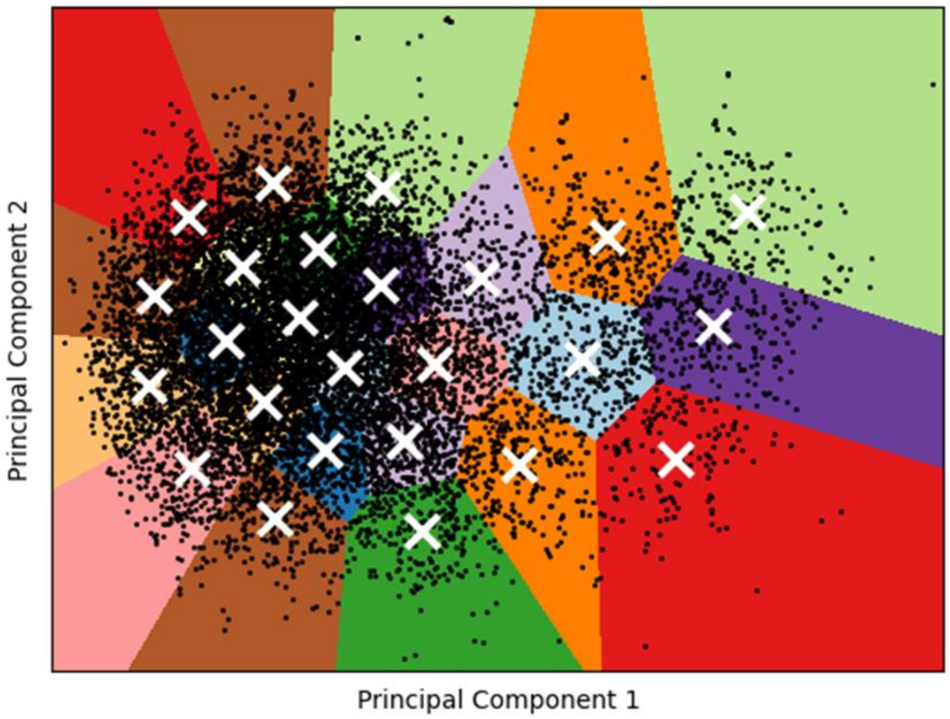
Principal Component 2
Principal Component 1

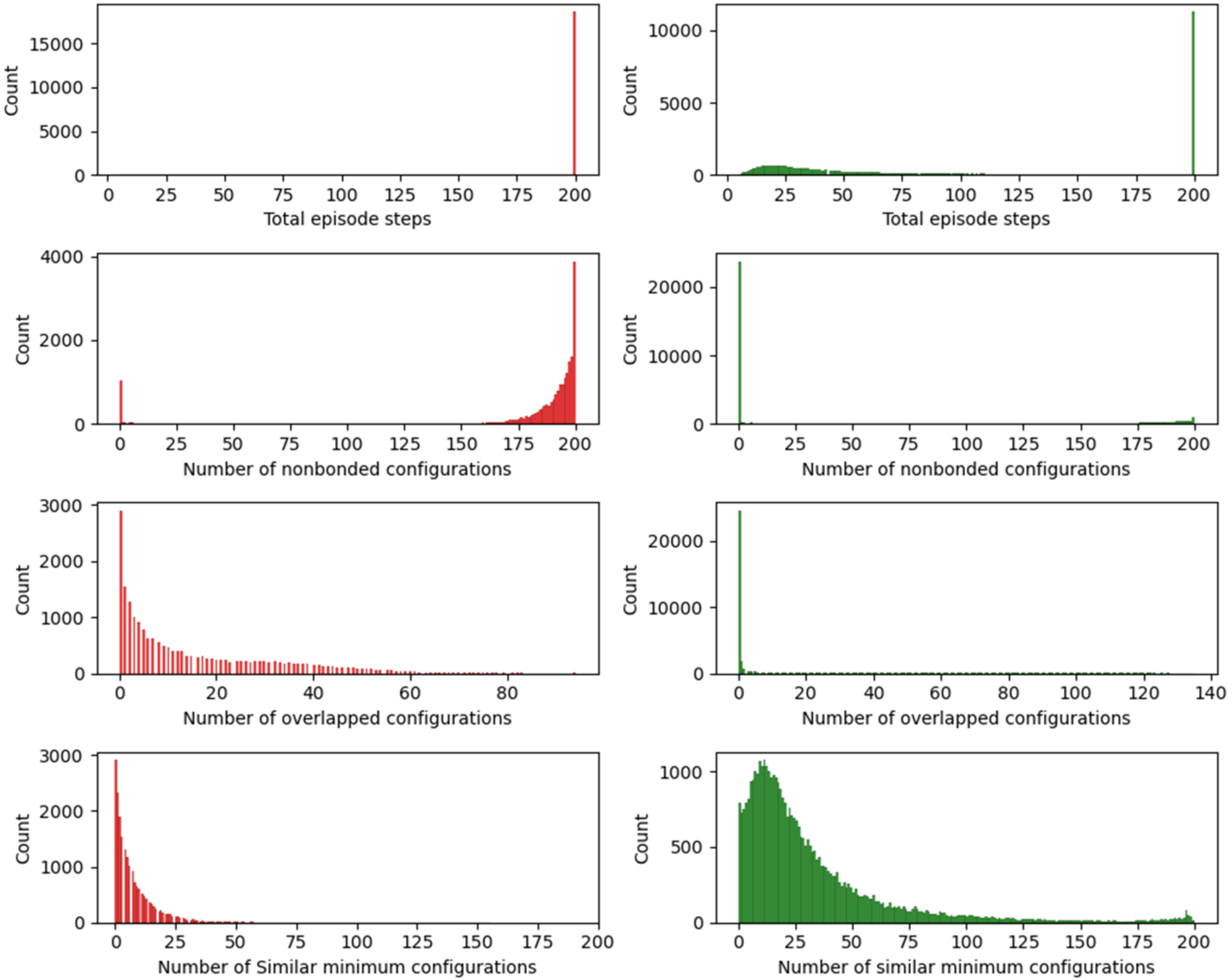
Count
Total episode steps
Number of nonbonded configurations
Number of overlapped configurations
Number of Similar minimum configurations
Number of similar minimum configurations

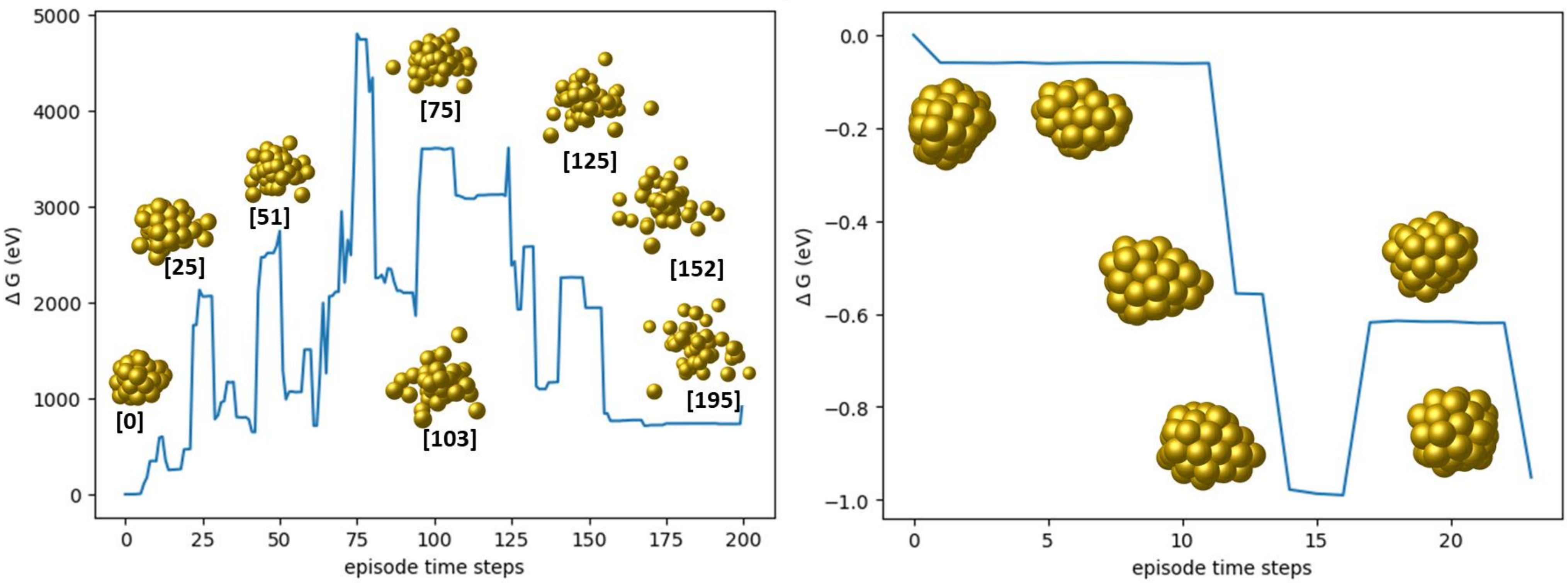

5000
4000
3000
2000
1000
0
Δ G (eV)
0
25
50
75
100
125
150
175
200
episode time steps
[0]
[25]
[51]
[75]
[103]
[125]
[152]
[195]
0.0
−0.2
−0.4
−0.6
−0.8
−1.0
Δ G (eV)
0
5
10
15
20
episode time steps

2. Ni_{35}

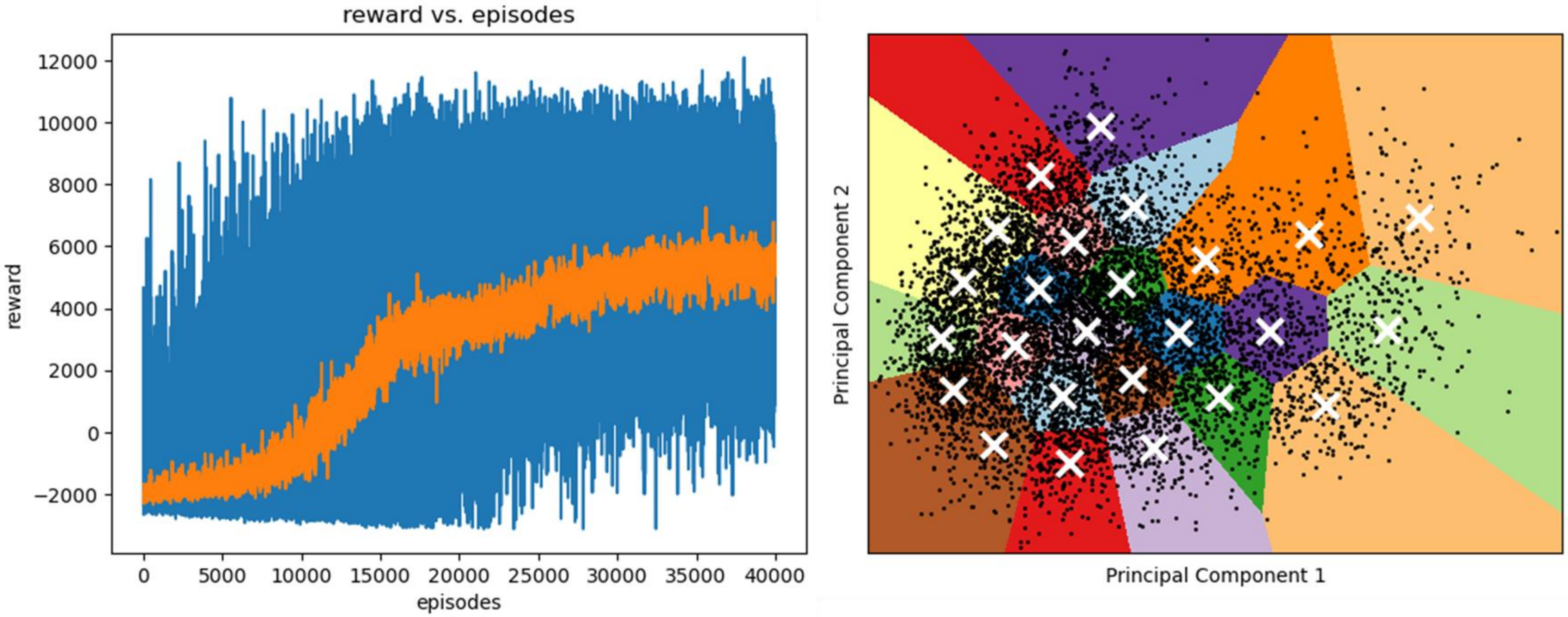
reward vs. episodes
reward
episodes
12000
10000
8000
6000
4000
2000
0
−2000
0
5000
10000
15000
20000
25000
30000
35000
40000
Principal Component 2
Principal Component 1

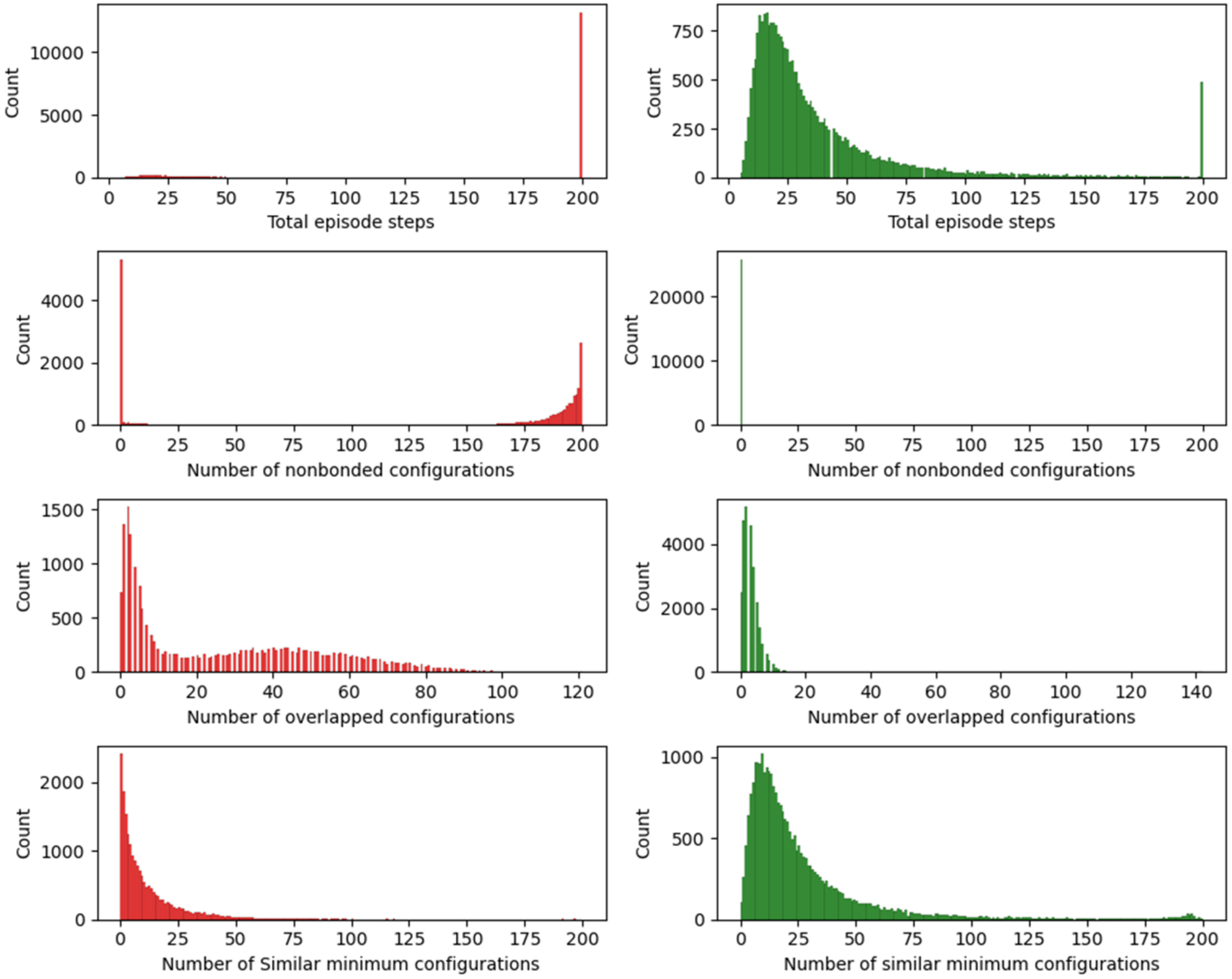

Count
Total episode steps
Number of nonbonded configurations
Number of overlapped configurations
Number of Similar minimum configurations
Number of similar minimum configurations

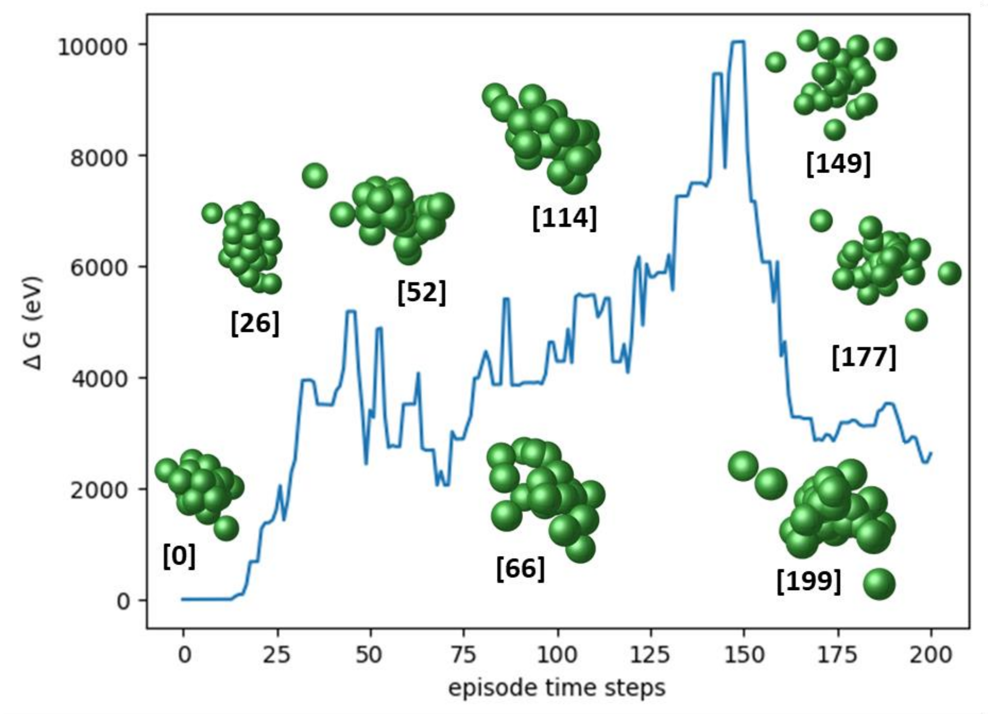
ΔG (eV)
episode time steps
[0]
[26]
[52]
[66]
[114]
[149]
[177]
[199]

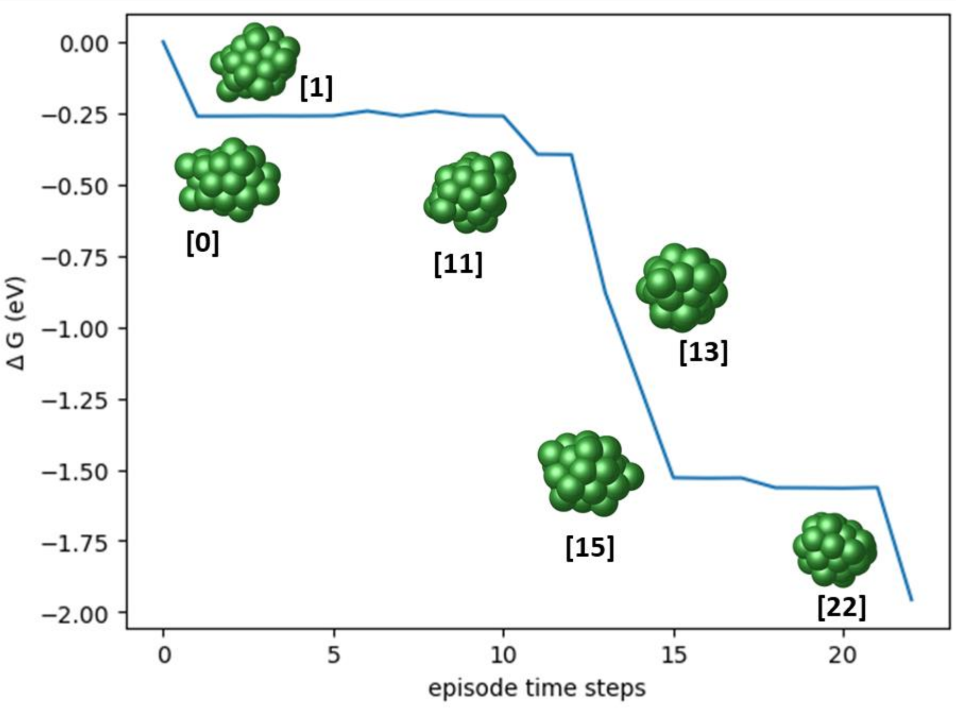
ΔG (eV)
episode time steps
[0]
[1]
[11]
[13]
[15]
[22]

3. Pd_{27}

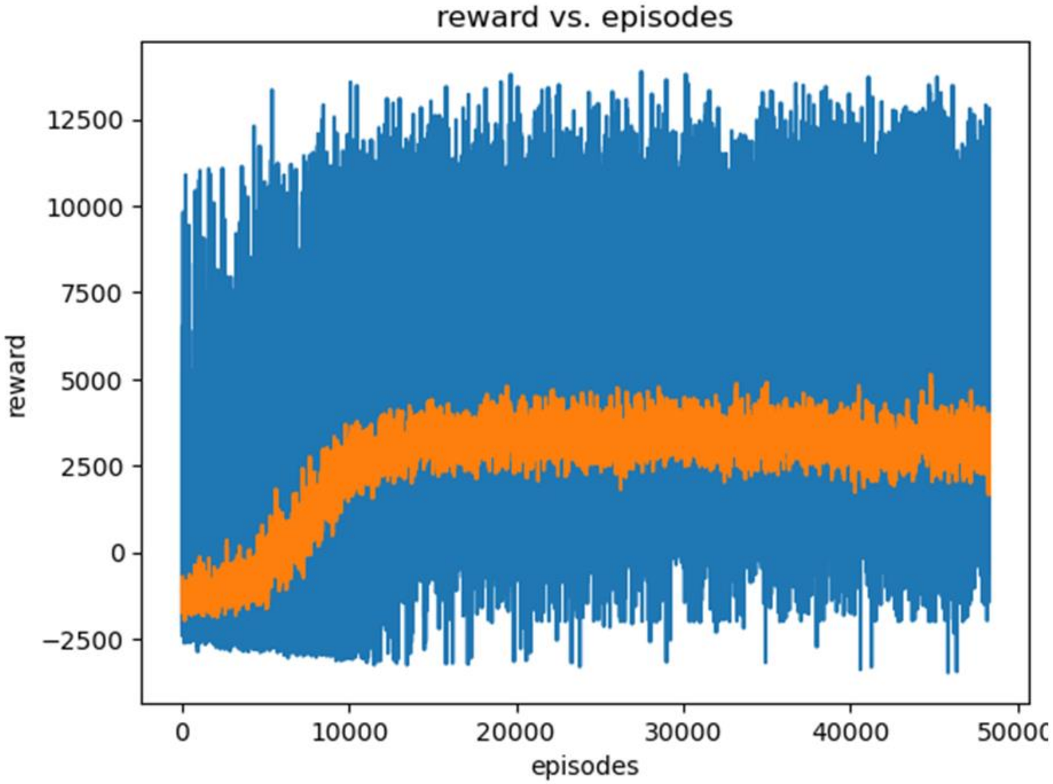
reward vs. episodes
reward
episodes
12500
10000
7500
5000
2500
0
−2500
0
10000
20000
30000
40000
50000

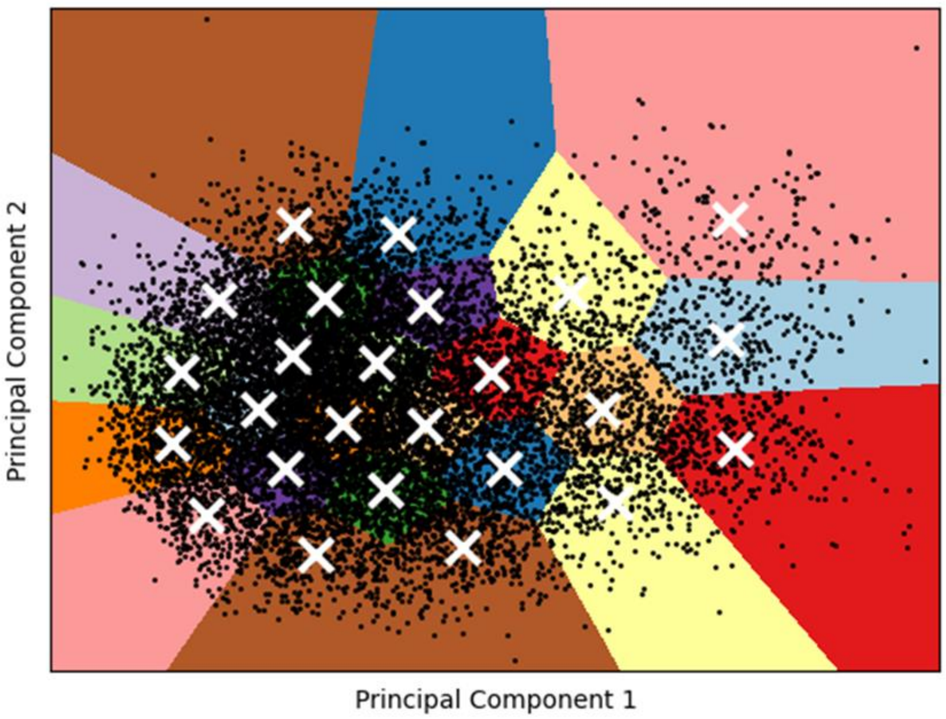
Principal Component 2
Principal Component 1

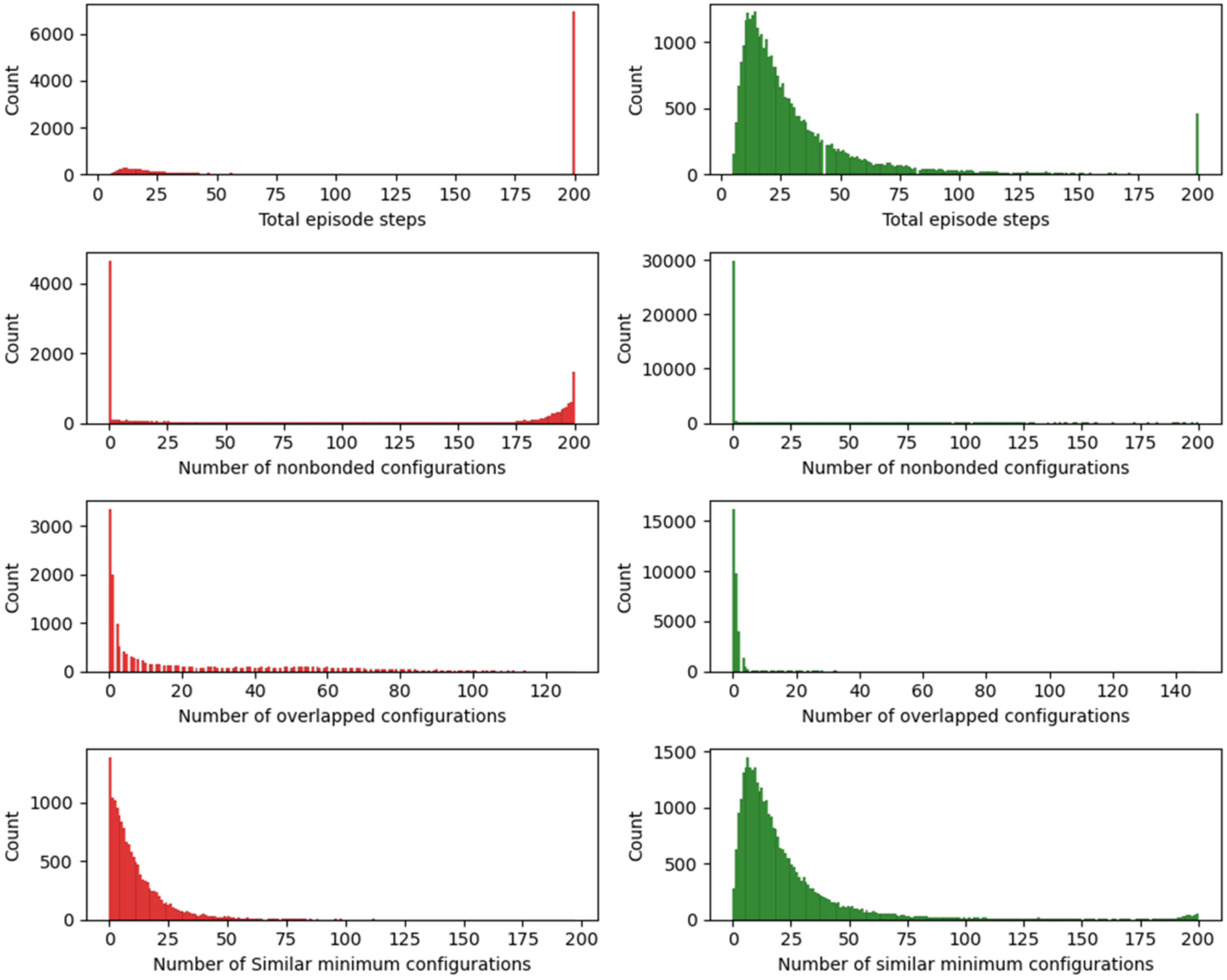
Count
6000
4000
2000
0
0 25 50 75 100 125 150 175 200
Total episode steps
Count
1000
500
0
0 25 50 75 100 125 150 175 200
Total episode steps
Count
4000
2000
0
0 25 50 75 100 125 150 175 200
Number of nonbonded configurations
Count
30000
20000
10000
0
0 25 50 75 100 125 150 175 200
Number of nonbonded configurations
Count
3000
2000
1000
0
0 20 40 60 80 100 120
Number of overlapped configurations
Count
15000
10000
5000
0
0 20 40 60 80 100 120 140
Number of overlapped configurations
Count
1000
500
0
0 25 50 75 100 125 150 175 200
Number of Similar minimum configurations
Count
1500
1000
500
0
0 25 50 75 100 125 150 175 200
Number of similar minimum configurations

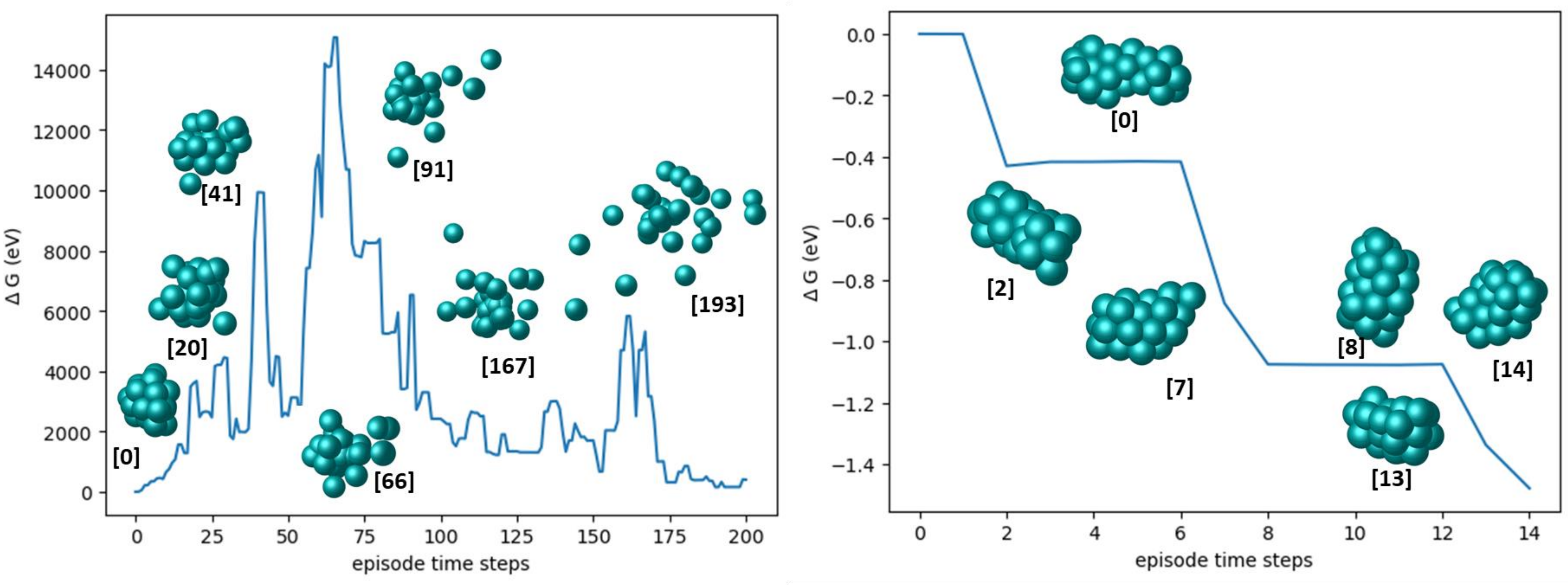

ΔG (eV)
episode time steps
[0]
[20]
[41]
[66]
[91]
[167]
[193]
ΔG (eV)
episode time steps
[0]
[2]
[7]
[8]
[13]
[14]

4. $Au_{18}Cu_{16}$

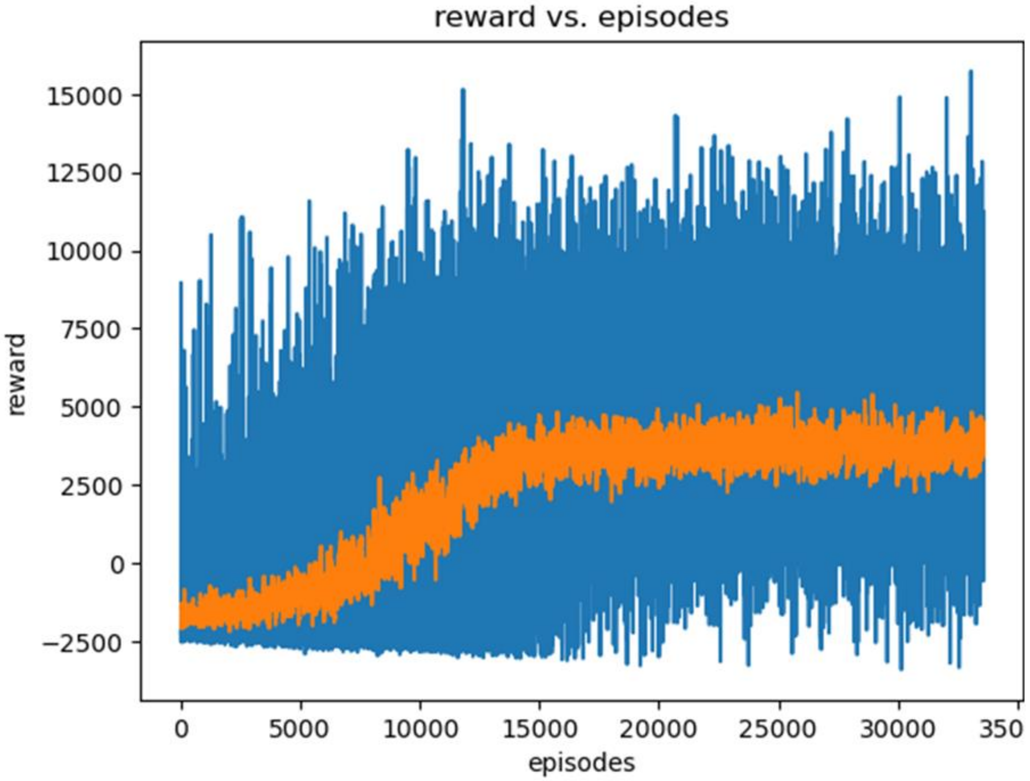
reward vs. episodes
15000
12500
10000
7500
5000
2500
0
−2500
reward
0
5000
10000
15000
20000
25000
30000
350
episodes

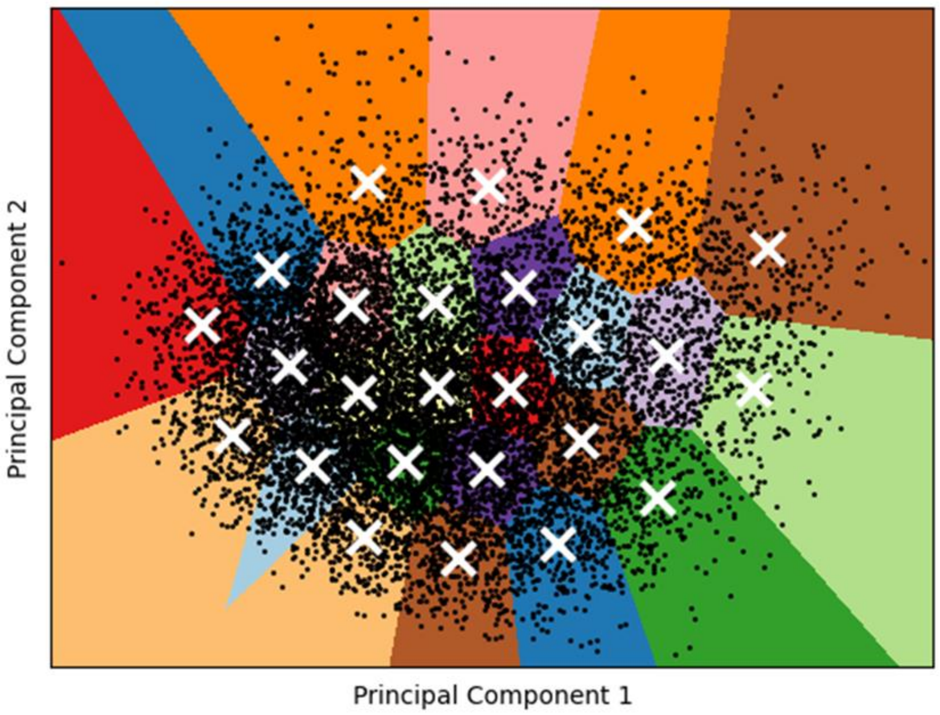
Principal Component 2
Principal Component 1

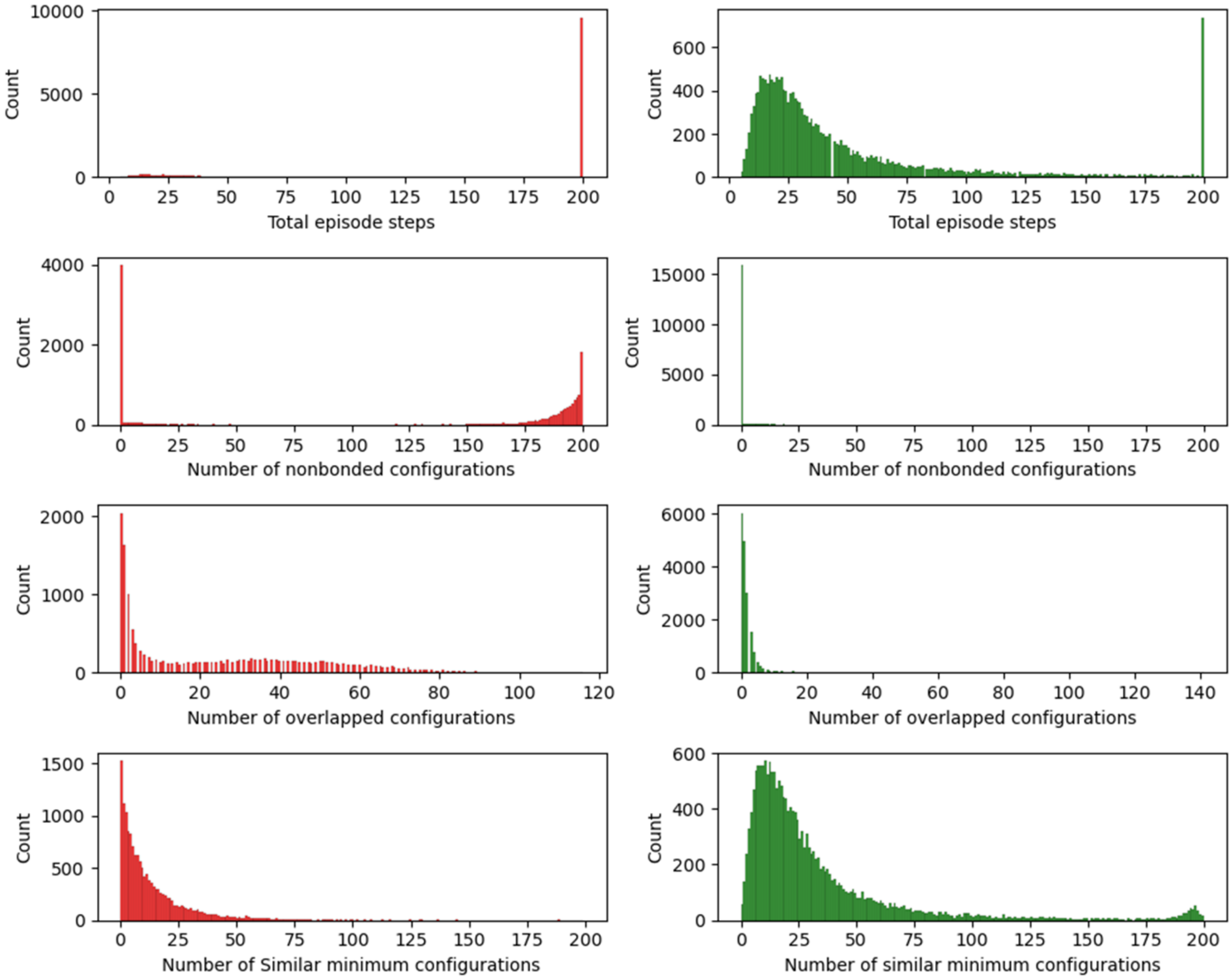
Count
Total episode steps
Count
Total episode steps
Count
Number of nonbonded configurations
Count
Number of nonbonded configurations
Count
Number of overlapped configurations
Count
Number of overlapped configurations
Count
Number of Similar minimum configurations
Count
Number of similar minimum configurations

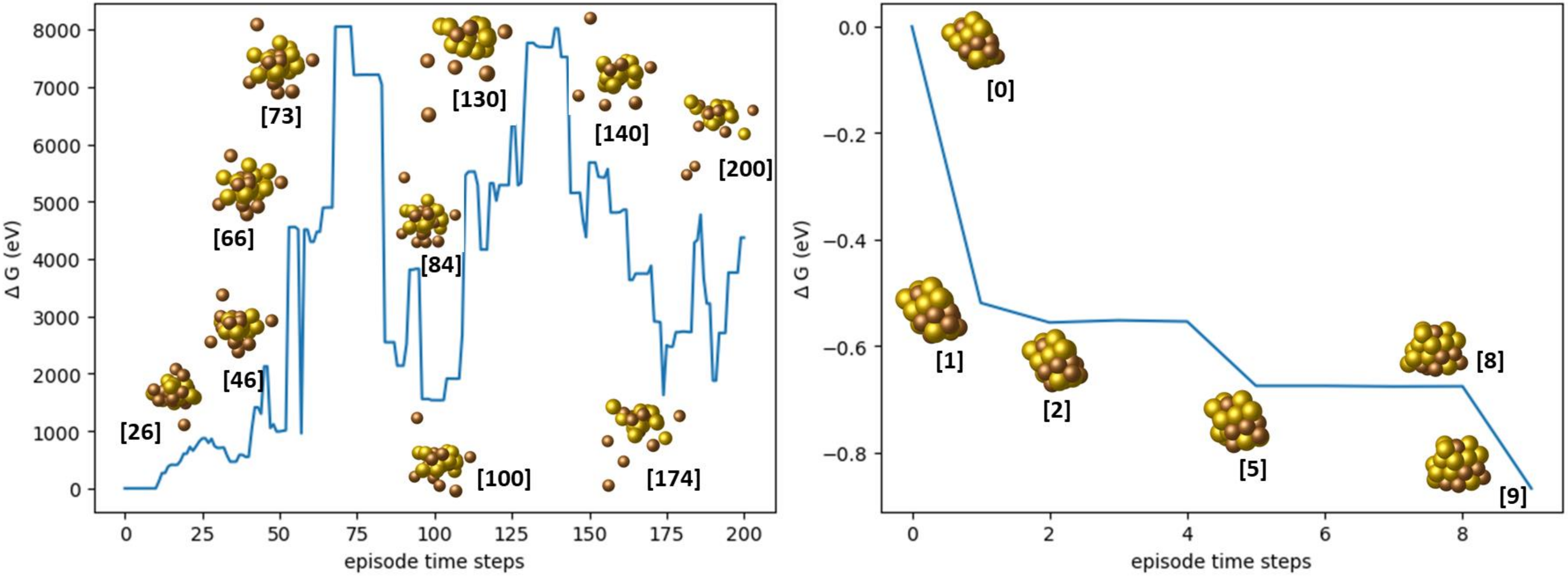

8000
7000
6000
5000
4000
3000
2000
1000
0
Δ G (eV)
0
25
50
75
100
125
150
175
200
episode time steps
[26]
[46]
[66]
[73]
[84]
[100]
[130]
[140]
[174]
[200]
0.0
−0.2
−0.4
−0.6
−0.8
Δ G (eV)
0
2
4
6
8
episode time steps
[0]
[1]
[2]
[5]
[8]
[9]

5. $Ag_{20}Au_{15}$

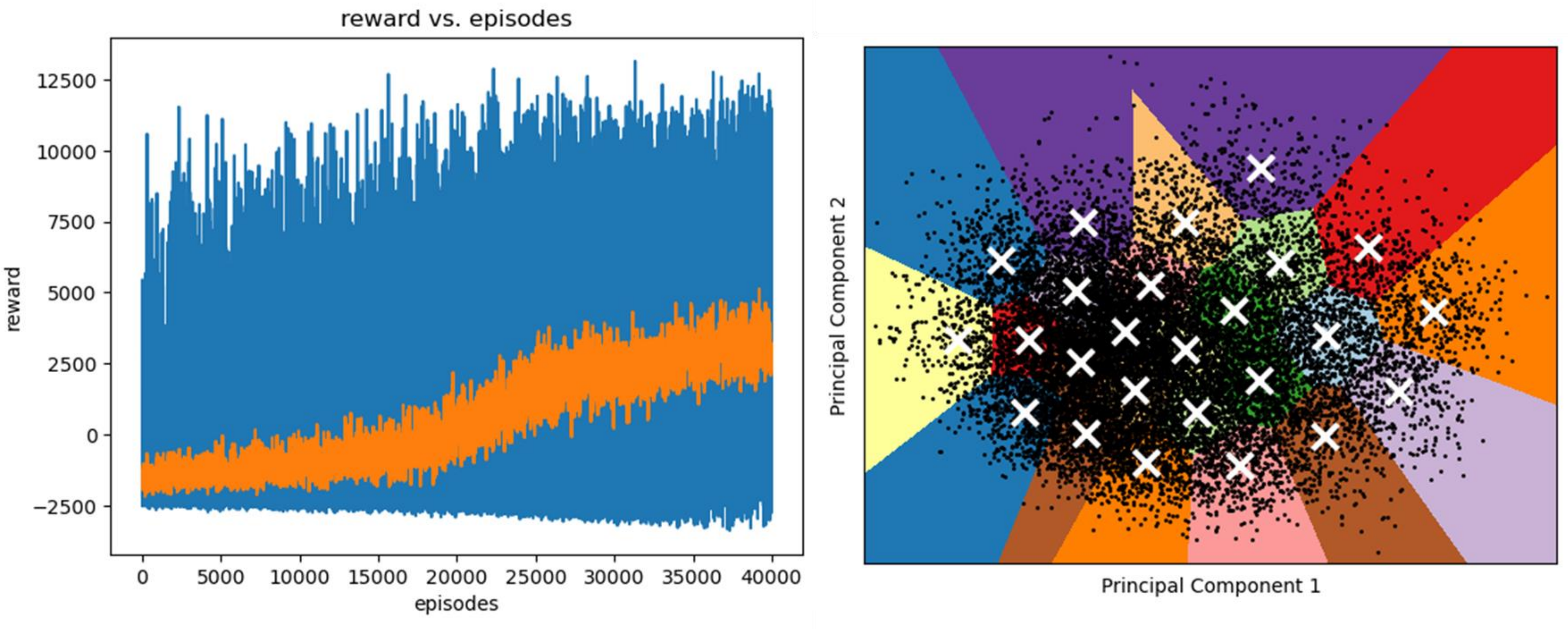
reward vs. episodes
reward
12500
10000
7500
5000
2500
0
−2500
0
5000
10000
15000
20000
25000
30000
35000
40000
episodes
Principal Component 2
Principal Component 1

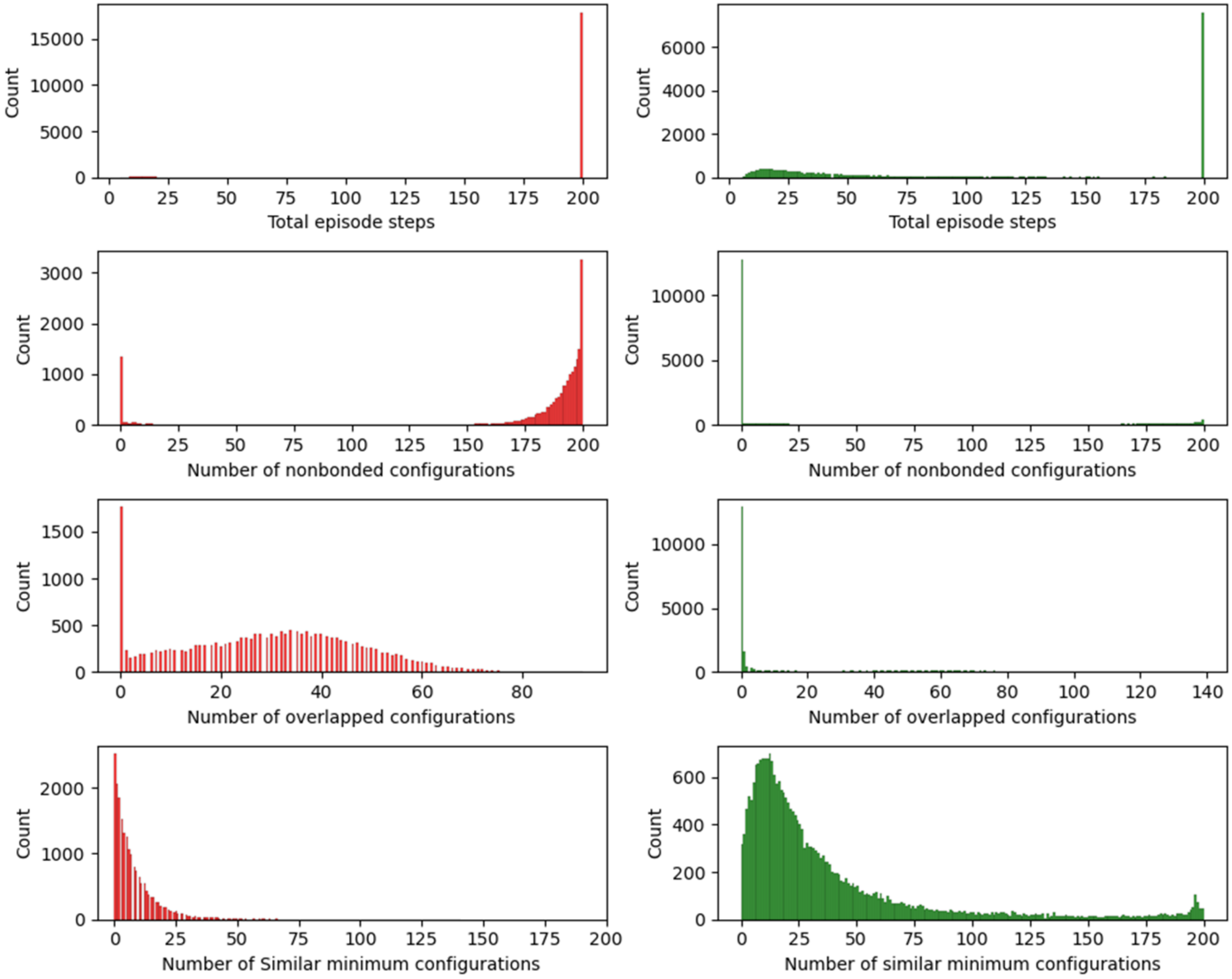
Count
15000
10000
5000
0
0 25 50 75 100 125 150 175 200
Total episode steps
Count
6000
4000
2000
0
0 25 50 75 100 125 150 175 200
Total episode steps
Count
3000
2000
1000
0
0 25 50 75 100 125 150 175 200
Number of nonbonded configurations
Count
10000
5000
0
0 25 50 75 100 125 150 175 200
Number of nonbonded configurations
Count
1500
1000
500
0
0 20 40 60 80
Number of overlapped configurations
Count
10000
5000
0
0 20 40 60 80 100 120 140
Number of overlapped configurations
Count
2000
1000
0
0 25 50 75 100 125 150 175 200
Number of Similar minimum configurations
Count
600
400
200
0
0 25 50 75 100 125 150 175 200
Number of similar minimum configurations

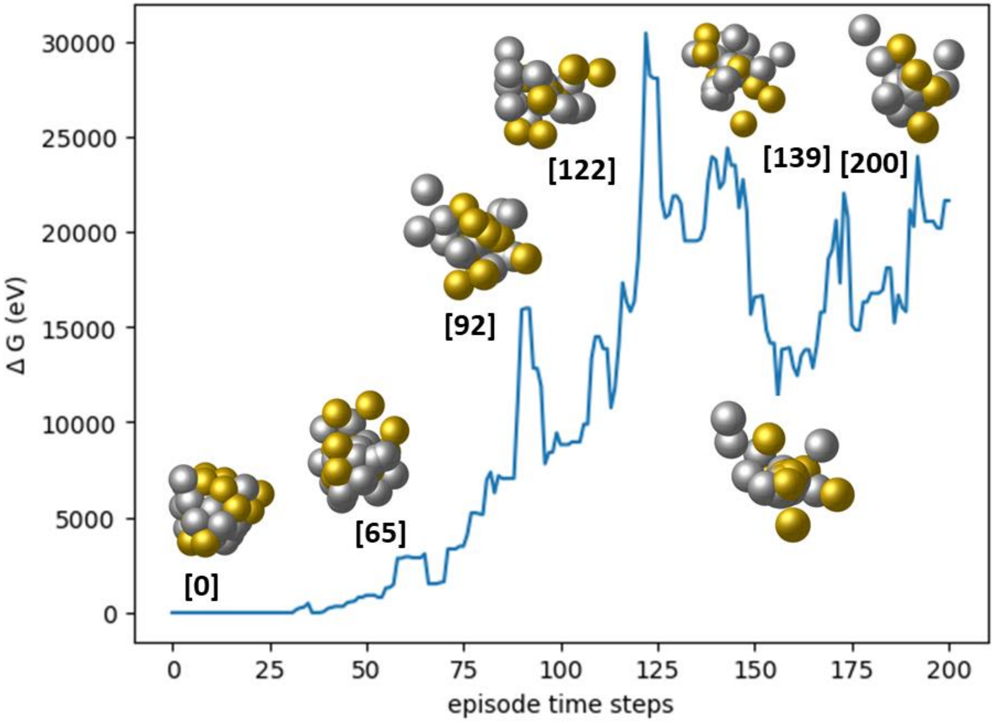
30000
25000
20000
15000
10000
5000
0
Δ G (eV)
0
25
50
75
100
125
150
175
200
episode time steps
[0]
[65]
[92]
[122]
[139]
[200]

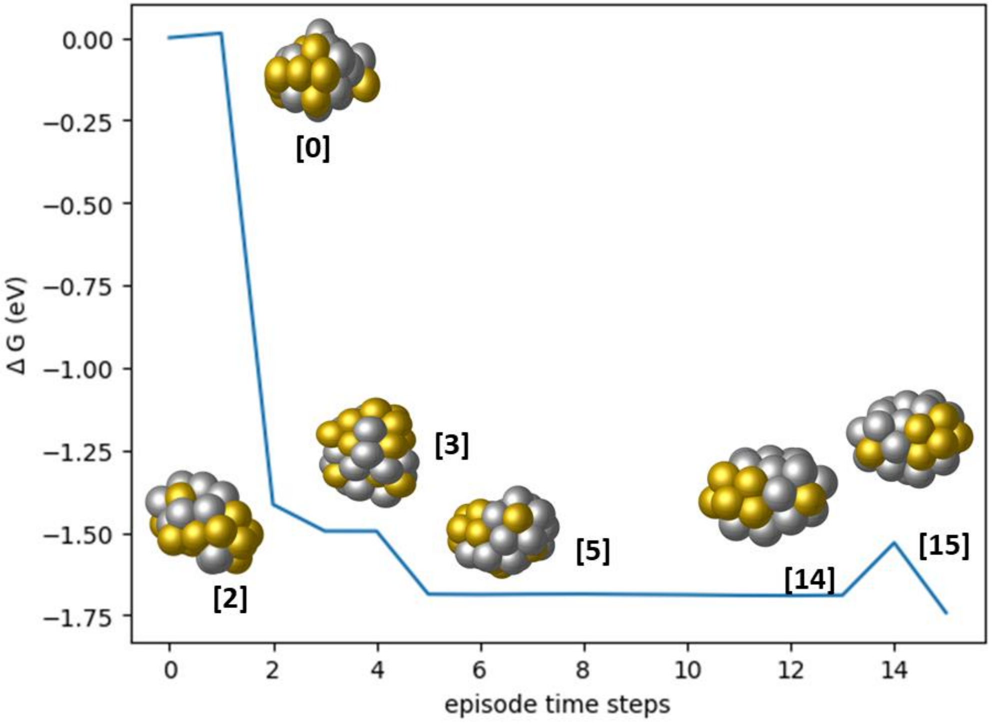
0.00
−0.25
−0.50
−0.75
−1.00
−1.25
−1.50
−1.75
Δ G (eV)
0
2
4
6
8
10
12
14
episode time steps
[0]
[2]
[3]
[5]
[14]
[15]

6. $Cu_{15}Pd_{15}$

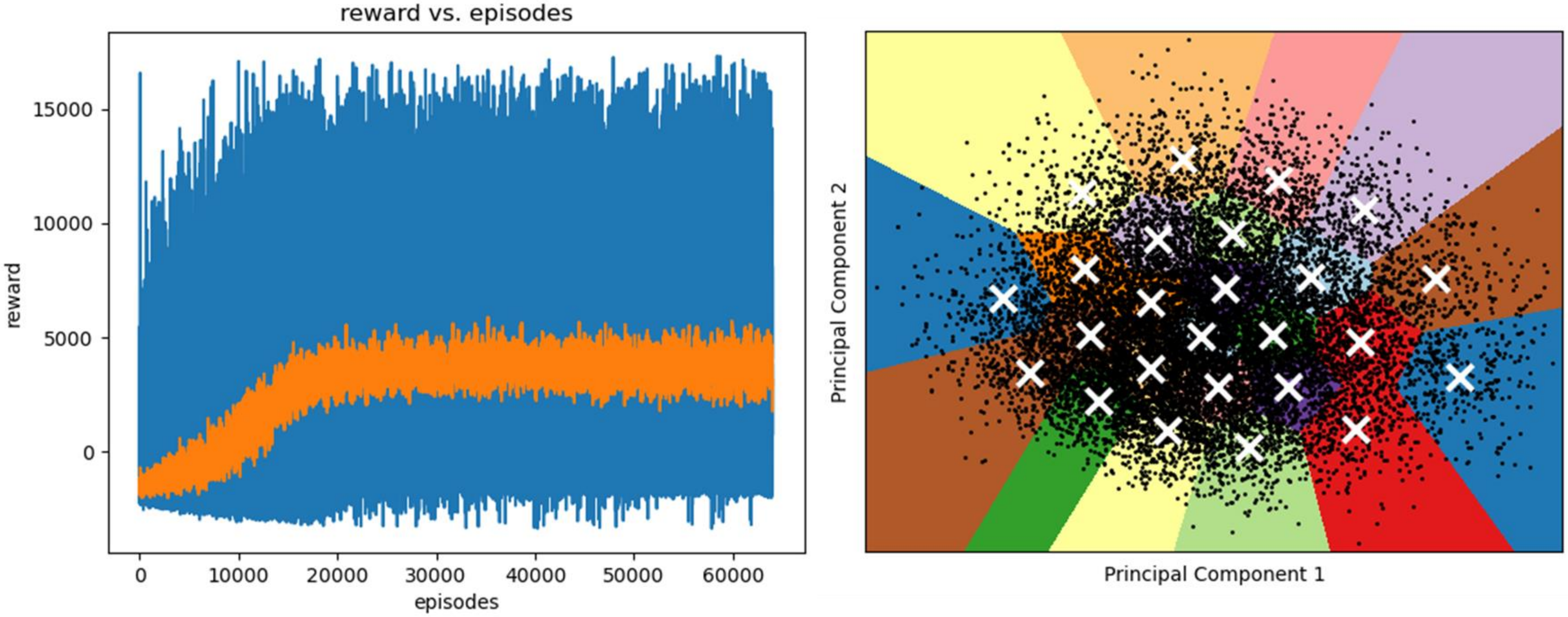
reward vs. episodes
reward
15000
10000
5000
0
0
10000
20000
30000
40000
50000
60000
episodes
Principal Component 2
Principal Component 1

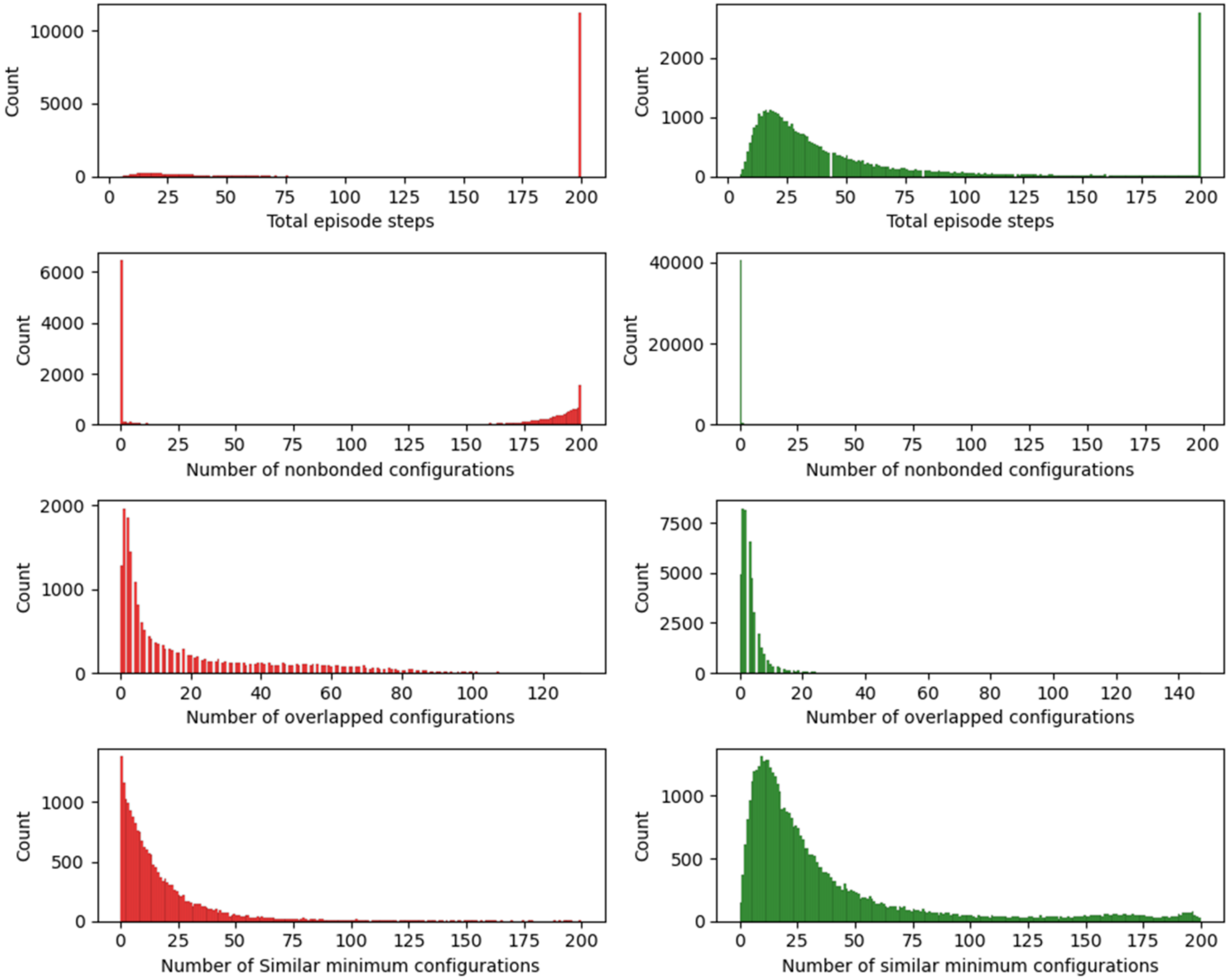
Count
Total episode steps
Number of nonbonded configurations
Number of overlapped configurations
Number of Similar minimum configurations
Number of similar minimum configurations

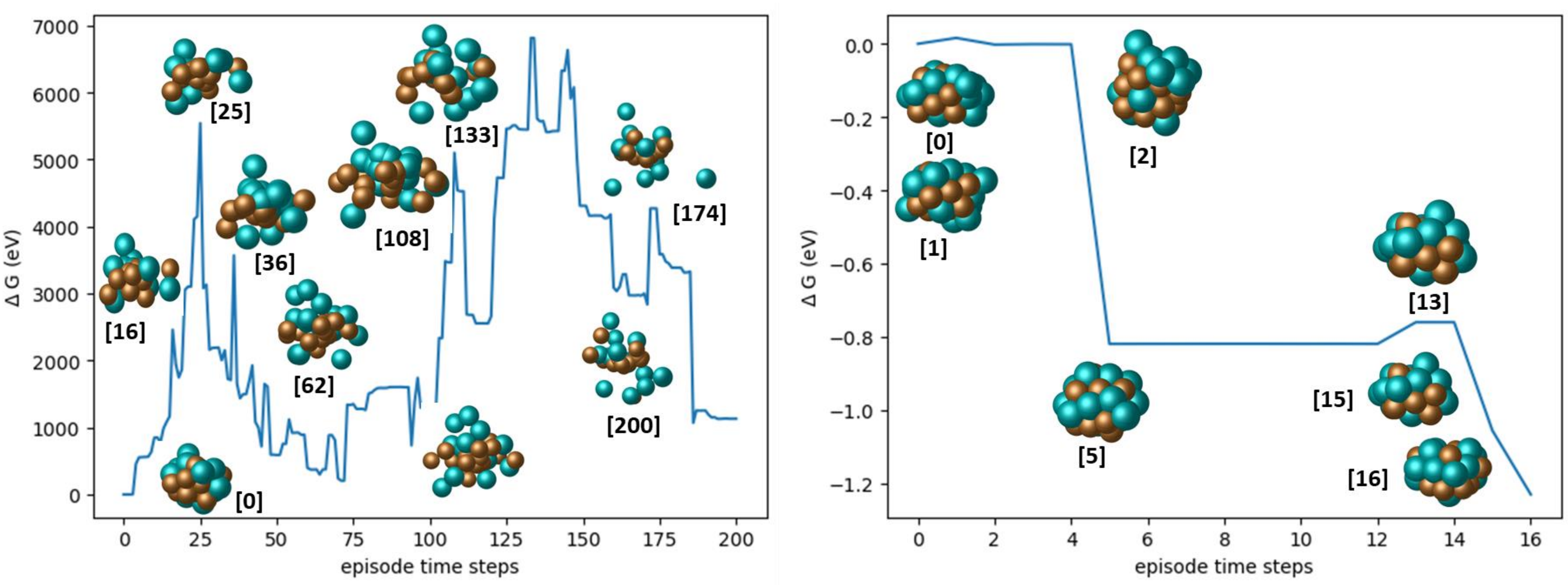

Δ G (eV)
7000
6000
5000
4000
3000
2000
1000
0
0
25
50
75
100
125
150
175
200
episode time steps
[25]
[133]
[174]
[36]
[108]
[16]
[62]
[200]
[0]
Δ G (eV)
0.0
−0.2
−0.4
−0.6
−0.8
−1.0
−1.2
0
2
4
6
8
10
12
14
16
episode time steps
[0]
[2]
[1]
[13]
[5]
[15]
[16]

7. $Cu_{22}Ni_{20}$

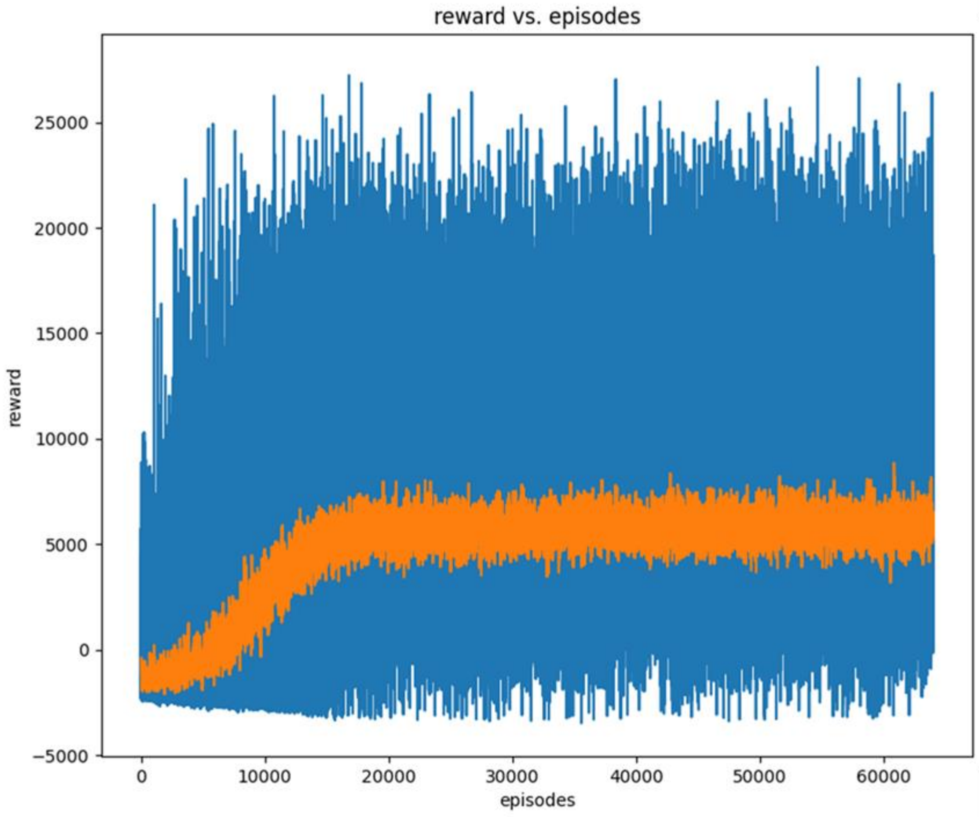
reward vs. episodes
reward
episodes
25000
20000
15000
10000
5000
0
−5000
0
10000
20000
30000
40000
50000
60000

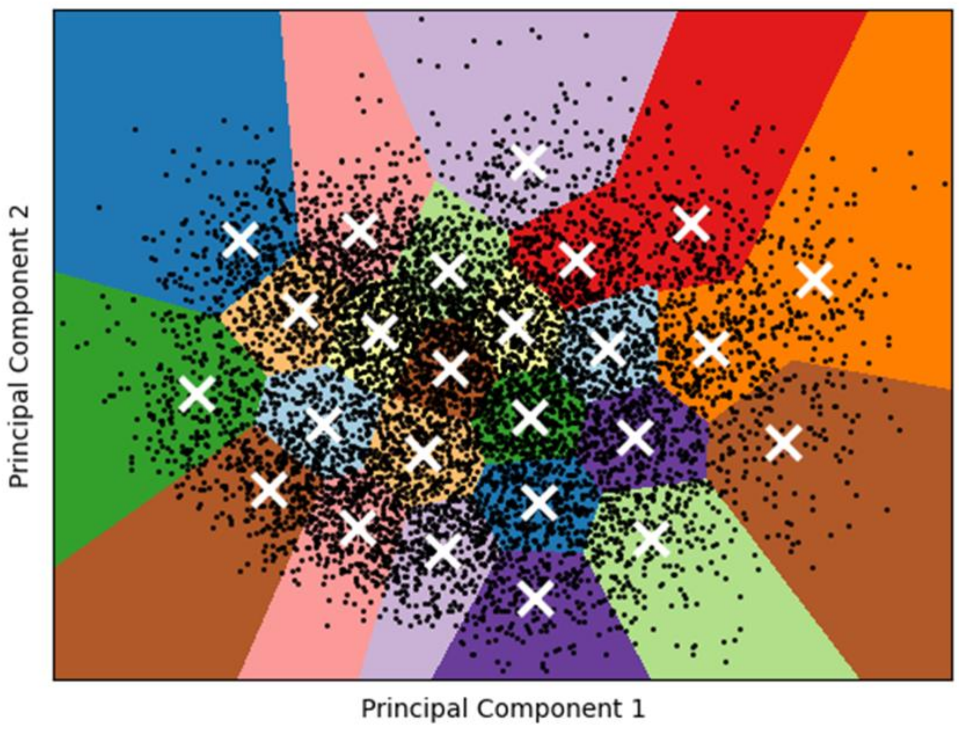
Principal Component 2
Principal Component 1

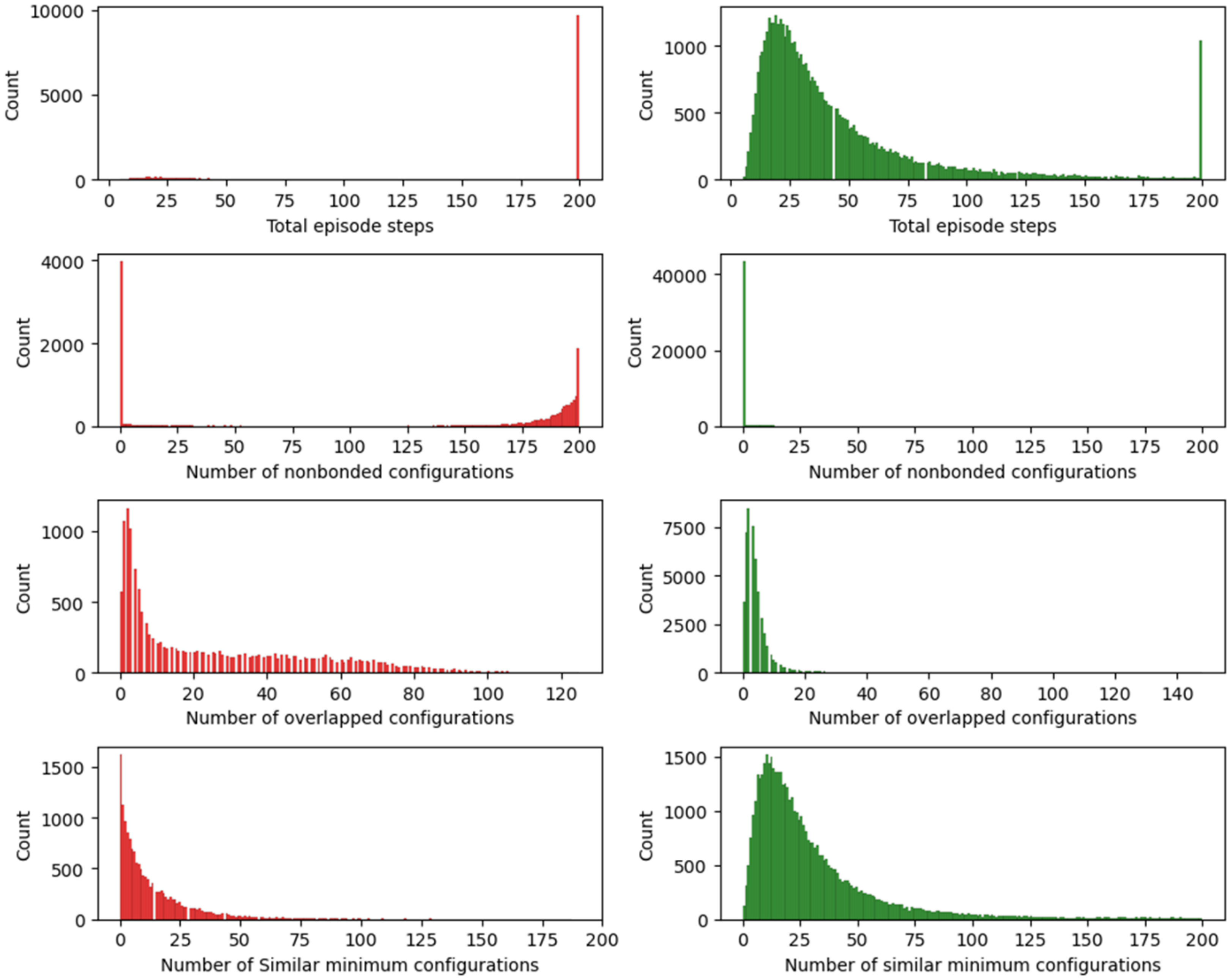
Count
Total episode steps
Number of nonbonded configurations
Number of overlapped configurations
Number of Similar minimum configurations
Number of similar minimum configurations

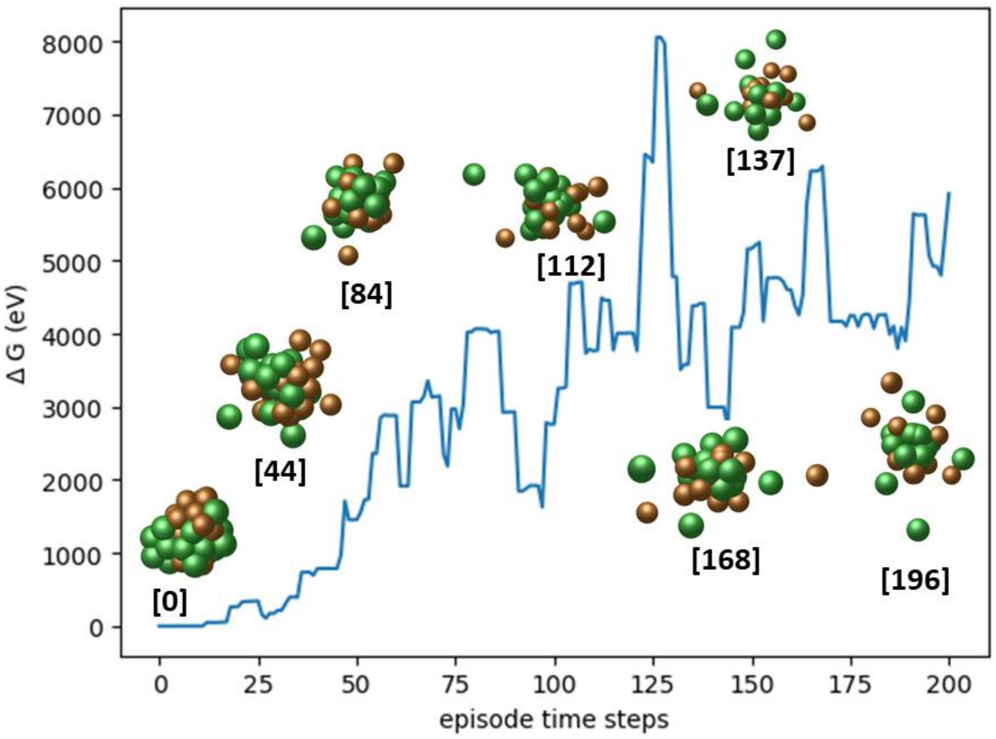

8000
7000
6000
5000
4000
3000
2000
1000
0
Δ G (eV)
0
25
50
75
100
125
150
175
200
episode time steps
[0]
[44]
[84]
[112]
[137]
[168]
[196]

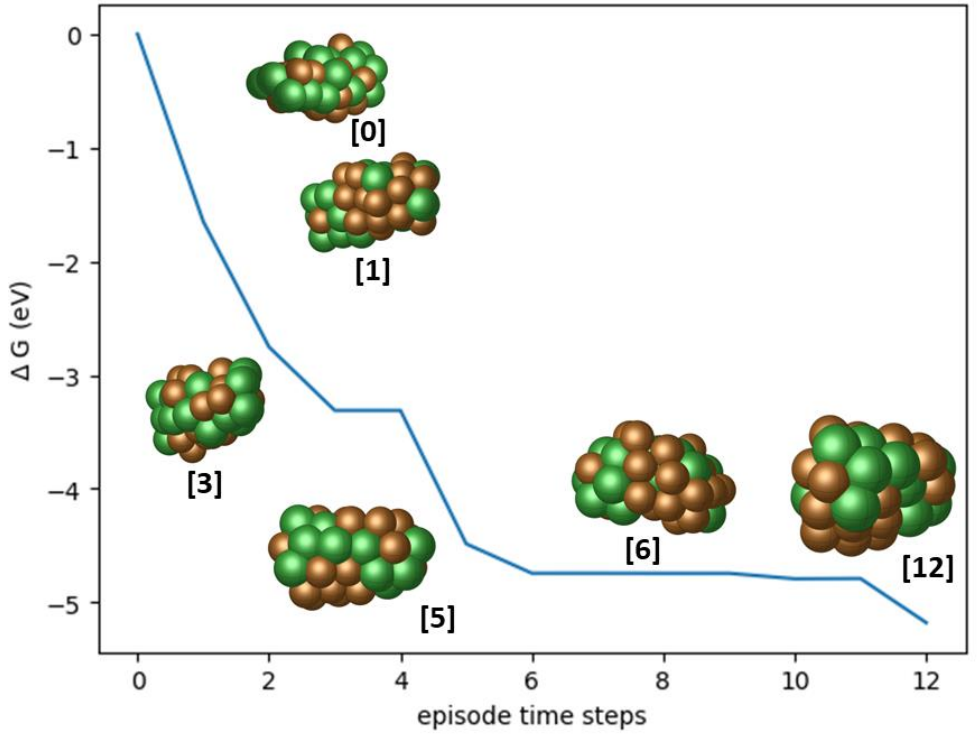

0
−1
−2
−3
−4
−5
Δ G (eV)
0
2
4
6
8
10
12
episode time steps
[0]
[1]
[3]
[5]
[6]
[12]

8. $Au_{12}Pd_{15}$

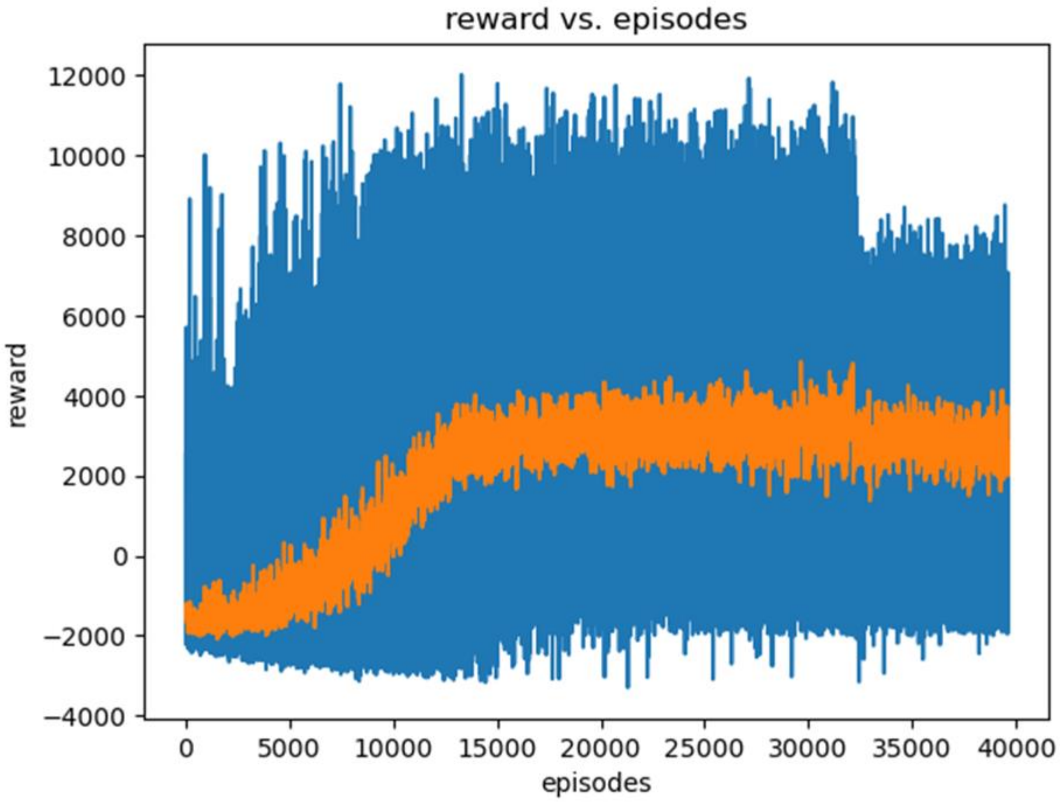
reward vs. episodes
reward
episodes
12000
10000
8000
6000
4000
2000
0
−2000
−4000
0
5000
10000
15000
20000
25000
30000
35000
40000

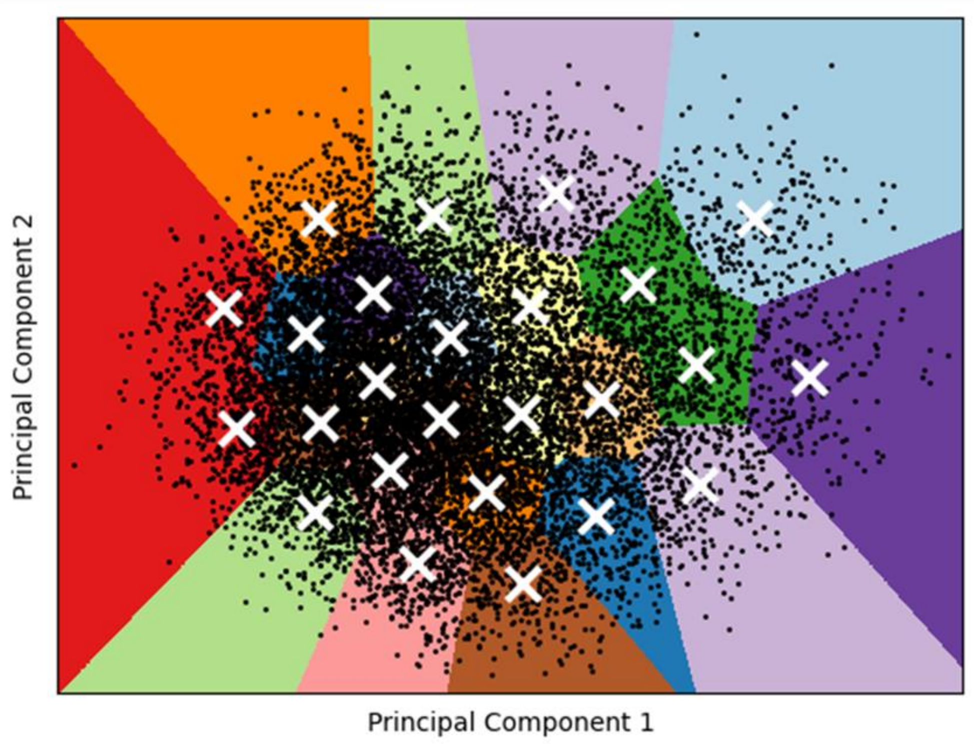
Principal Component 2
Principal Component 1

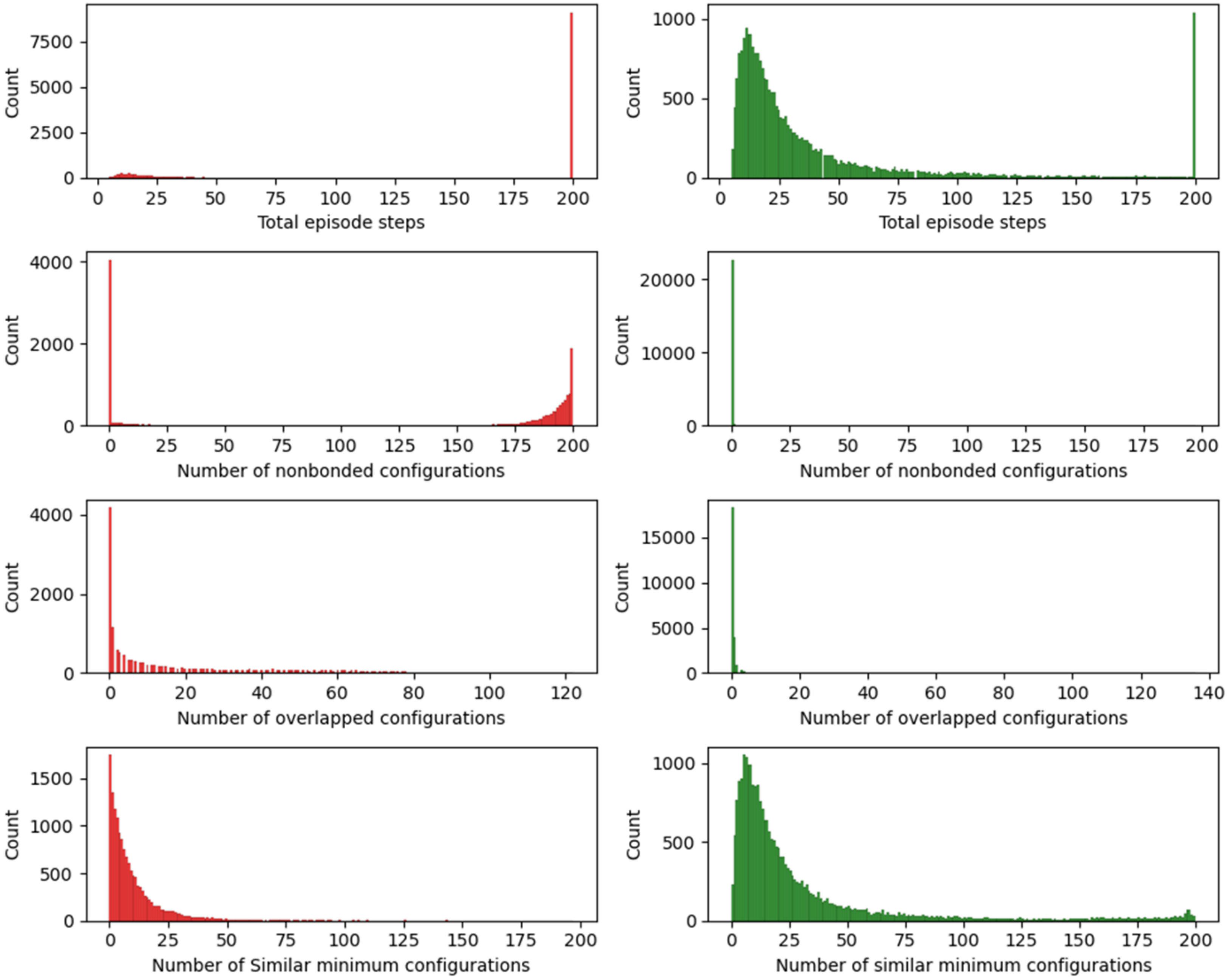
Count
7500
5000
2500
0
0 25 50 75 100 125 150 175 200
Total episode steps
Count
1000
500
0
0 25 50 75 100 125 150 175 200
Total episode steps
Count
4000
2000
0
0 25 50 75 100 125 150 175 200
Number of nonbonded configurations
Count
20000
10000
0
0 25 50 75 100 125 150 175 200
Number of nonbonded configurations
Count
4000
2000
0
0 20 40 60 80 100 120
Number of overlapped configurations
Count
15000
10000
5000
0
0 20 40 60 80 100 120 140
Number of overlapped configurations
Count
1500
1000
500
0
0 25 50 75 100 125 150 175 200
Number of Similar minimum configurations
Count
1000
500
0
0 25 50 75 100 125 150 175 200
Number of similar minimum configurations

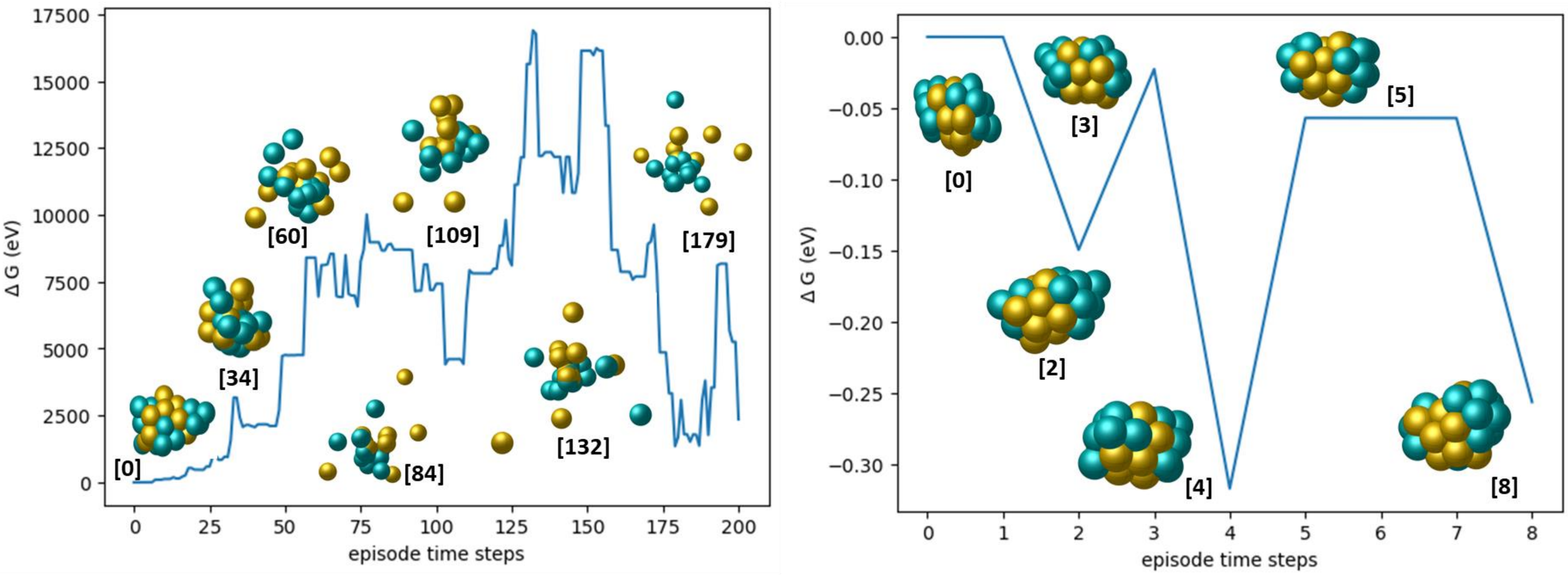
17500
15000
12500
10000
7500
5000
2500
0
Δ G (eV)
0
25
50
75
100
125
150
175
200
episode time steps
[0]
[34]
[60]
[84]
[109]
[132]
[179]
0.00
−0.05
−0.10
−0.15
−0.20
−0.25
−0.30
Δ G (eV)
0
1
2
3
4
5
6
7
8
episode time steps
[0]
[2]
[3]
[4]
[5]
[8]

9. $Ni_{10}Pd_{13}$

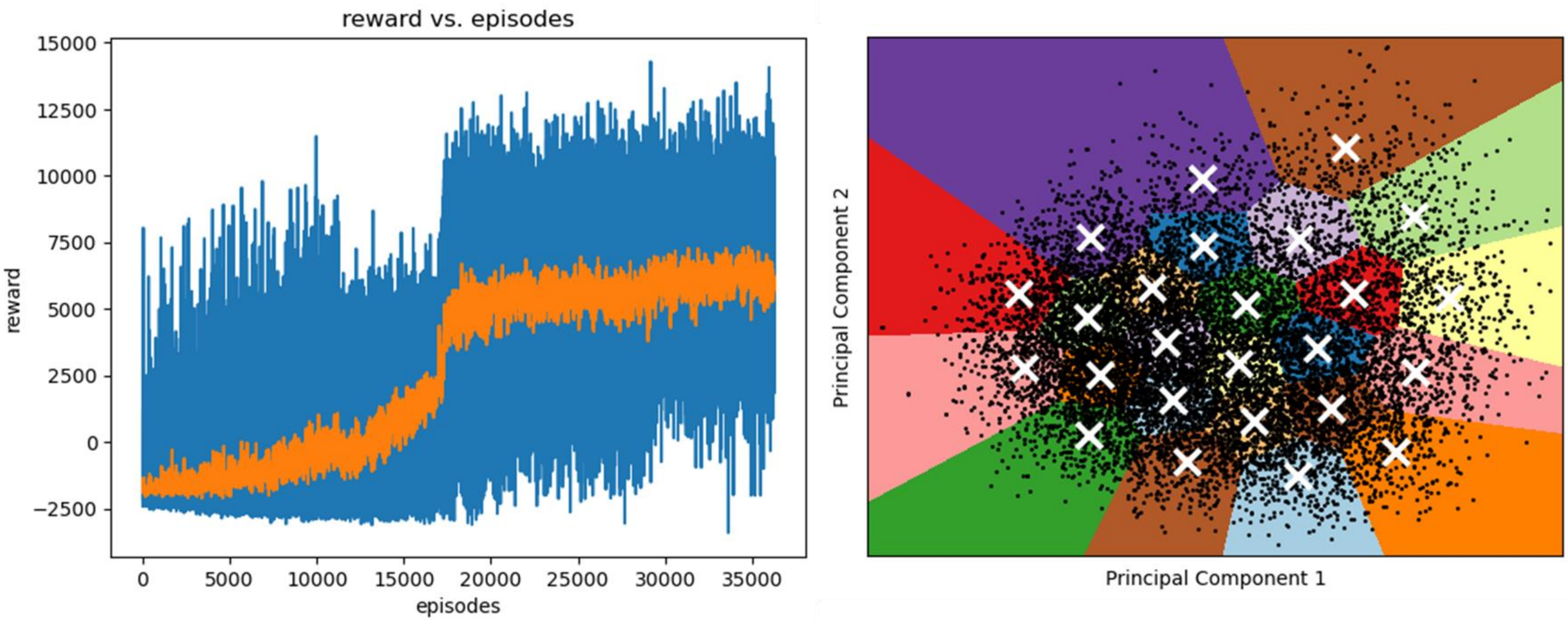
reward vs. episodes
15000
12500
10000
7500
5000
2500
0
−2500
reward
0
5000
10000
15000
20000
25000
30000
35000
episodes
Principal Component 2
Principal Component 1

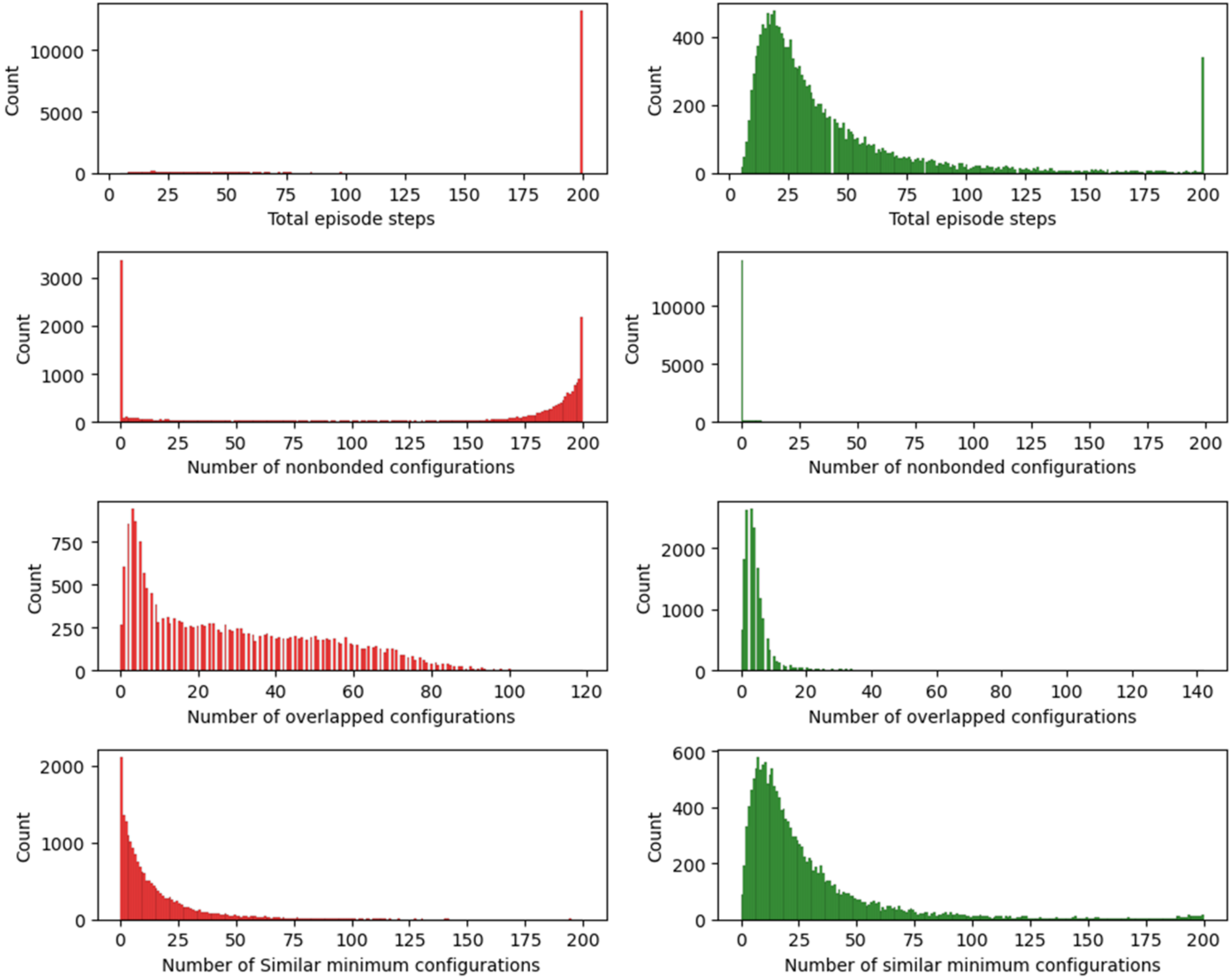
Count
Total episode steps
Number of nonbonded configurations
Number of overlapped configurations
Number of Similar minimum configurations
Number of similar minimum configurations

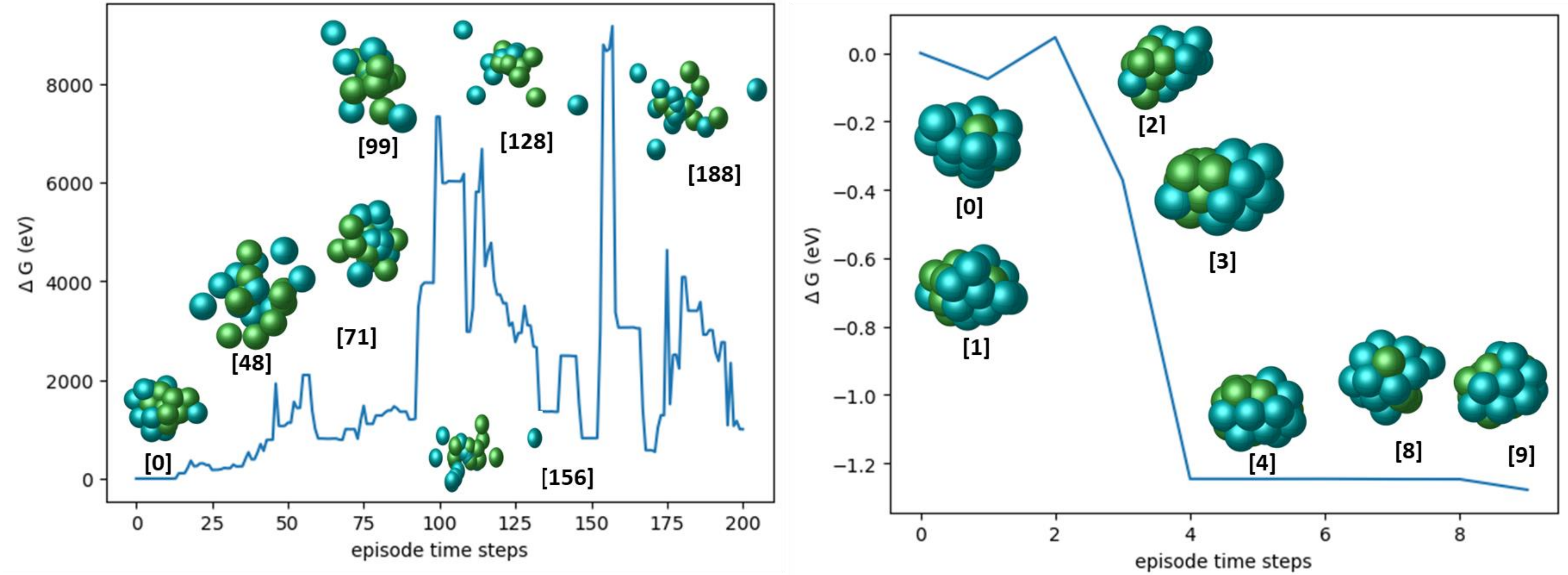

ΔG (eV)
episode time steps
[0]
[48]
[71]
[99]
[128]
[156]
[188]
[0]
[1]
[2]
[3]
[4]
[8]
[9]

10. $Cu_4Pd_5Ni_6$

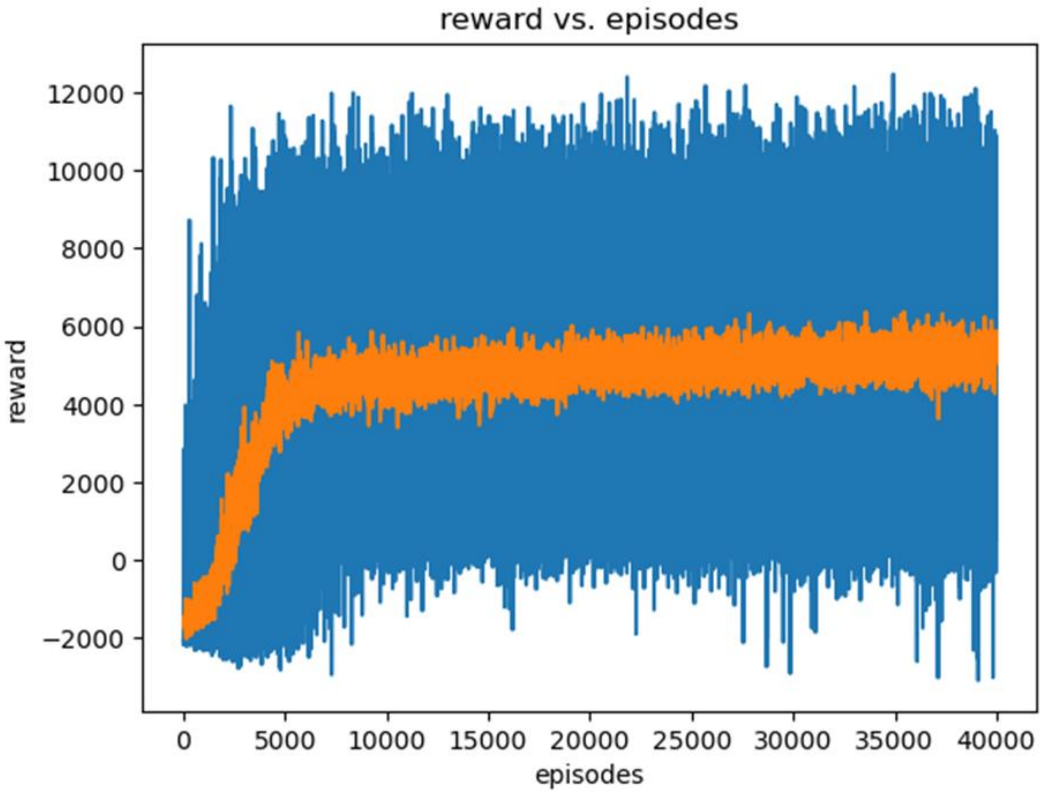

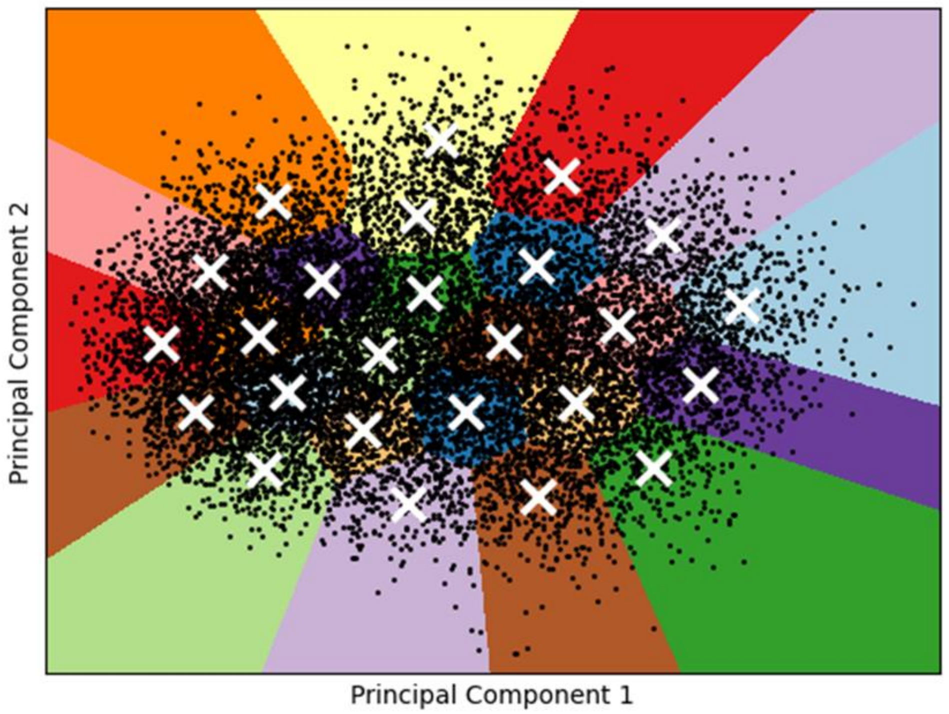

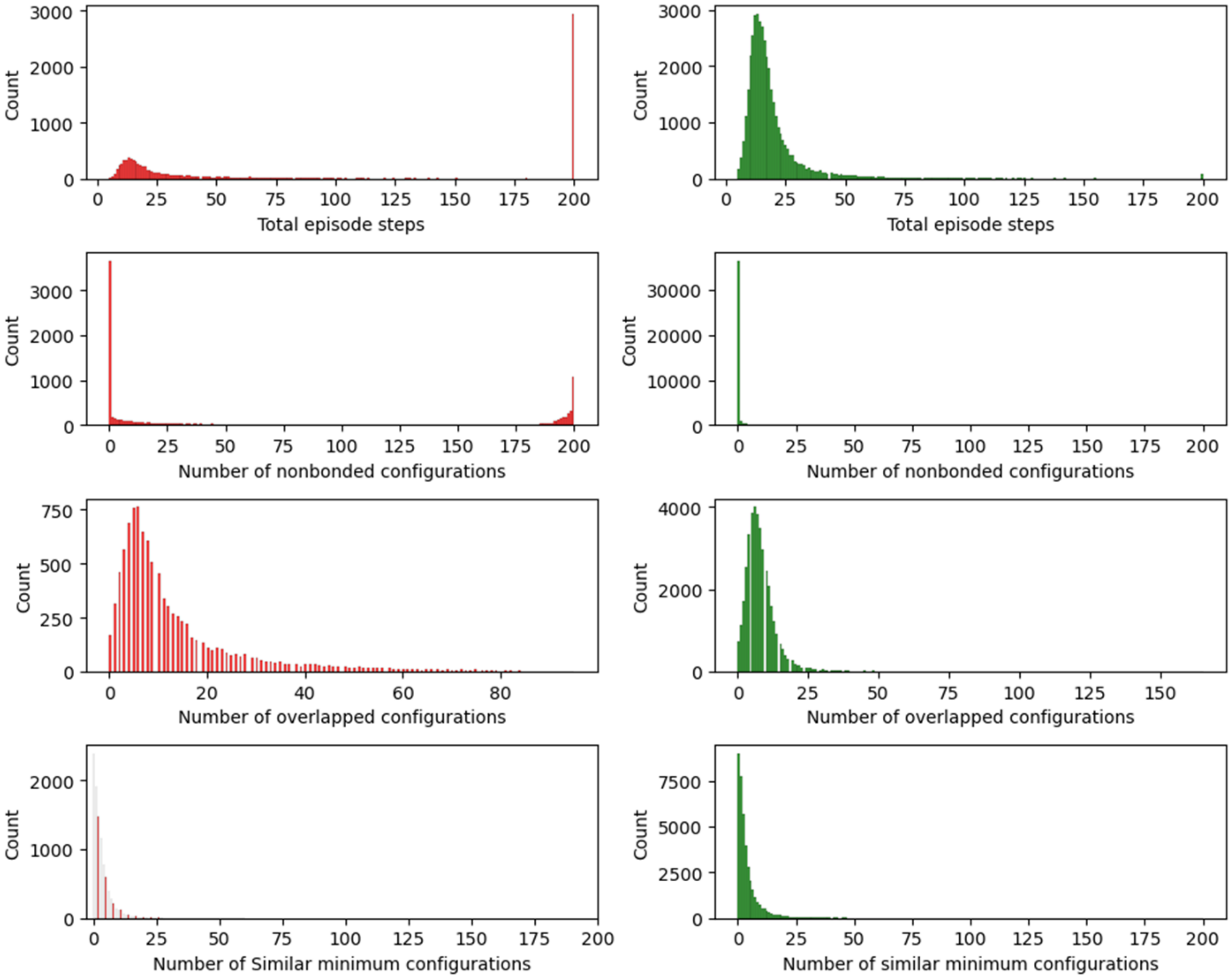

Count
Total episode steps
Count
Total episode steps
Count
Number of nonbonded configurations
Count
Number of nonbonded configurations
Count
Number of overlapped configurations
Count
Number of overlapped configurations
Count
Number of Similar minimum configurations
Count
Number of similar minimum configurations

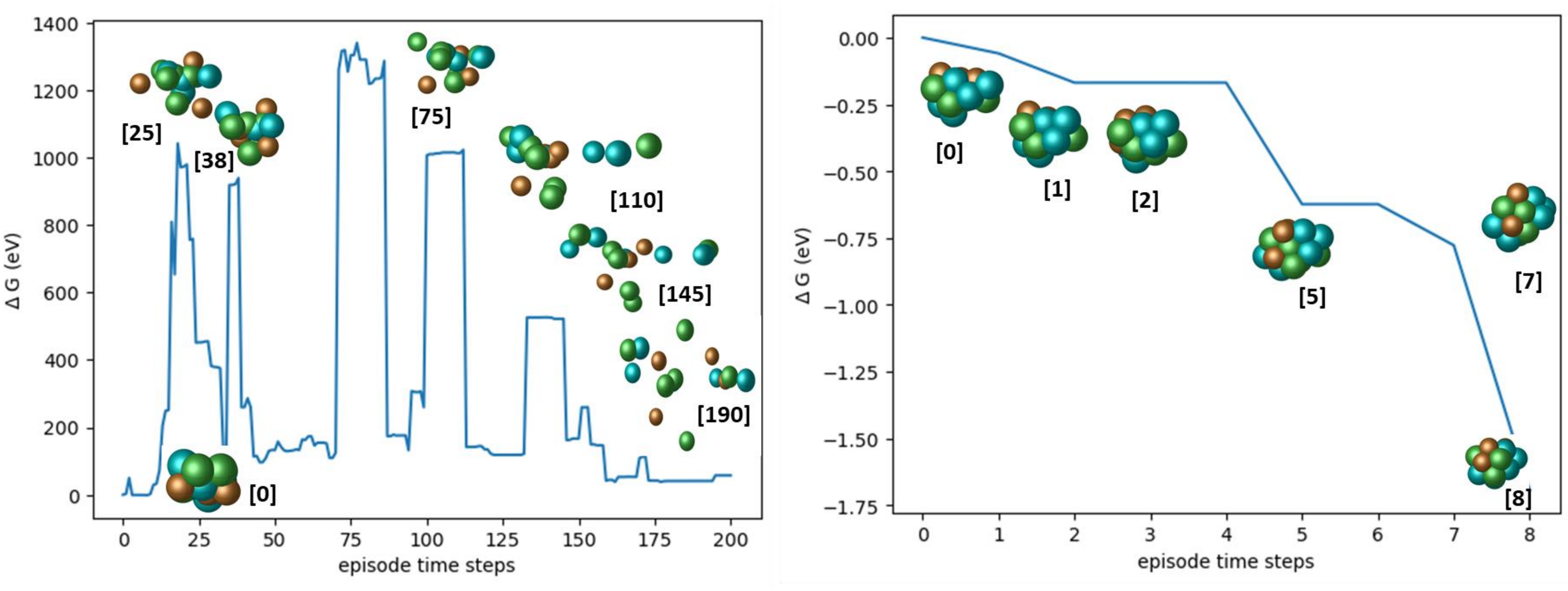

Δ G (eV)
episode time steps
[25]
[38]
[75]
[110]
[145]
[190]
[0]
[1]
[2]
[5]
[7]
[8]
